# Physical mapping of QTL for tuber yield, starch content and starch yield in tetraploid potato (*Solanum tuberosum* L.) by means of genome wide genotyping by sequencing and the 8.3 K SolCAP SNP array

**DOI:** 10.1186/s12864-017-3979-9

**Published:** 2017-08-22

**Authors:** Elske Maria Schönhals, Jia Ding, Enrique Ritter, Maria João Paulo, Nicolás Cara, Ekhard Tacke, Hans-Reinhard Hofferbert, Jens Lübeck, Josef Strahwald, Christiane Gebhardt

**Affiliations:** 1 Department of Plant Breeding and Genetics, Max Planck Institute for Plant Breeding Research, Cologne, Germany; 2 NEIKER, Vitoria-Gasteiz, Spain; 3 Biometris, Wageningen University, Wageningen, The Netherlands; 4 Bioplant, Ebstorf, Germany; 5 Böhm-Nordkartoffel Agrarproduktion GmbH & Co. OHG, Ebstorf, Germany; 6 SaKa-Pflanzenzucht GmbH & Co. KG, Windeby, Germany

**Keywords:** Potato (*Solanum tuberosum*), Tuber, Yield, Starch content, Complex trait, Genome wide genotyping, SNP, GWAS, Candidate gene

## Abstract

**Background:**

Tuber yield and starch content of the cultivated potato are complex traits of decisive importance for breeding improved varieties. Natural variation of tuber yield and starch content depends on the environment and on multiple, mostly unknown genetic factors. Dissection and molecular identification of the genes and their natural allelic variants controlling these complex traits will lead to the development of diagnostic DNA-based markers, by which precision and efficiency of selection can be increased (precision breeding).

**Results:**

Three case-control populations were assembled from tetraploid potato cultivars based on maximizing the differences between high and low tuber yield (TY), starch content (TSC) and starch yield (TSY, arithmetic product of TY and TSC). The case-control populations were genotyped by restriction-site associated DNA sequencing (RADseq) and the 8.3 k SolCAP SNP genotyping array. The allele frequencies of single nucleotide polymorphisms (SNPs) were compared between cases and controls. RADseq identified, depending on data filtering criteria, between 6664 and 450 genes with one or more differential SNPs for one, two or all three traits. Differential SNPs in 275 genes were detected using the SolCAP array. A genome wide association study using the SolCAP array on an independent, unselected population identified SNPs associated with tuber starch content in 117 genes. Physical mapping of the genes containing differential or associated SNPs, and comparisons between the two genome wide genotyping methods and two different populations identified genome segments on all twelve potato chromosomes harboring one or more quantitative trait loci (QTL) for TY, TSC and TSY.

**Conclusions:**

Several hundred genes control tuber yield and starch content in potato. They are unequally distributed on all potato chromosomes, forming clusters between 0.5–4 Mbp width. The largest fraction of these genes had unknown function, followed by genes with putative signalling and regulatory functions. The genetic control of tuber yield and starch content is interlinked. Most differential SNPs affecting both traits had antagonistic effects: The allele increasing TY decreased TSC and vice versa. Exceptions were 89 SNP alleles which had synergistic effects on TY, TSC and TSY. These and the corresponding genes are primary targets for developing diagnostic markers.

**Electronic supplementary material:**

The online version of this article (doi:10.1186/s12864-017-3979-9) contains supplementary material, which is available to authorized users.

## Background

Crop yield is essential in agriculture and therefore a major selection criterion in any crop breeding program. New cultivars with higher yield per area unit are required for producing enough food for a growing world population under limited availability of arable land. Other than in cereals, a high portion of the total yield of root and tuber crops (root or tuber weight per plant, per plot or area unit) consists of water. In case of the cultivated potato (*Solanum tuberosum* L.), the dry matter content of the tubers ranges from about 18 to 26% of the total yield. The main component is the glucose polymer starch [1] which accounts for approximately 80% of the dry matter. Next to starch, the sugars sucrose, glucose and fructose make up between 0.7% and 10% of the fresh weight [2]. Starch and sugars are interconverted in dormant tubers in response to environmental signals such as the storage temperature [3]. The tuber starch content (TSC) in percent of the total tuber weight is an important quality measure. It has an optimal range for different end uses of the potato crop such as direct consumption (10–17%), processing (14–20%) and industrial starch production (up to 25%). Tuber starch yield (TSY), that is the arithmetic product of the total yield and the percentage of starch, is a particularly relevant parameter for the production of industrial potato starch for multiple purposes [4]. Tuber yield (TY), starch content (TSC) and starch yield (TSY) are therefore decisive characters for the development of new potato varieties for different end uses.

The natural variation of tuber yield and starch content, and consequently of starch yield, depends on multiple genetic and environmental factors. The development of stably high yielding cultivars with optimal tuber starch content and several other quality criteria requires multiple year and location trials. Tuber starch content is reliably estimated by determining the tuber specific gravity via the under water weight, which is linear correlated with dry matter and starch content [5]. The assessment of tuber yield by weighing is simple but unreliable in the early stages of selection due to an insufficient number of tubers for conducting replicated field trials. Sufficient tuber numbers become available only after several years of vegetative multiplication. Dissection and molecular identification of the genetic factors that underly the natural variation of tuber yield and starch content facilitates the identification of superior allele combinations in parents as well as progeny by diagnostic DNA-based markers. Prudent use of diagnostic markers can increase the selection efficiency by identifying optimal parental combinations and reducing the number of progeny to be assessed in the field (precision breeding).

During the last twenty five years, linkage mapping of quantitative trait loci (QTL) for tuber yield and starch content (or specific gravity or dry matter), using various types of DNA-based markers, has been performed in a number of diploid and tetraploid, in intra- as well as interspecific potato families comprising between 50 and 230 full sib clones [6–11]. Irrespective of the marker density, which ranged from very low genome coverage with restriction fragment length polymorphism (RFLP) markers to high genome coverage with amplified fragment length polymorphism (AFLP) or single nucleotide polymorphism (SNP) markers, one to two QTL per chromosome and trait were distinguished. When tuber yield and starch content were evaluated in the same family, QTL for both traits were in several cases detected by the same markers, suggesting that the underlying genes are physically tightly linked, or alternatively, the same genes have pleiotropic effects on both traits, e.g. [6, 11]. Between two and eight QTL for tuber yield were mapped per family. The most consistent QTL for tuber yield in different genetic backgrounds were located on potato chromosomes I, II, V, VI and XII. In the case of tuber starch content, from one to sixteen QTL were dissected per family, which were distributed on all twelve potato chromosomes (summarized in [12]). The QTL with the largest effects on both tuber yield and starch content were located on potato chromosome V. The genetic resolution was low in these linkage studies, and the diagnostic power of QTL linked markers in genetic materials other than the experimental family used for QTL mapping was not further assessed in most cases.

Association mapping in populations of individuals related by descent increases the genetic resolution, as it takes advantage of recombination events over multiple meiotic generations in multiple parents [13]. Association mapping in populations of cultivars from advanced breeding programs has the advantage that markers can be obtained, which have diagnostic value in a wide genetic background suitable for variety development. In the cultivated potato, association analysis of tuber traits, among others tuber yield, starch content and starch yield, was performed during the last decade in populations comprising between 150 and 300 tetraploid varieties and breeding clones [12, 14–21]. Most of these association studies were based on the candidate gene approach, meaning that genotyping was targeted at known genes selected in a functional context. Populations were genotyped for DNA variants in genes that function in starch and sugar metabolism [12, 14–18, 20, 22], in enzymatic discoloration upon mechanical impact (tuber bruising) [18] and in genes resulting from comparative protein profiling of tubers with contrasting sugar content [21]. Thirty nine candidate loci showed DNA marker trait associations mostly with tuber starch content, several of those also with starch yield and only few with tuber yield. One association study in two variety panels used as markers more than three thousand untargeted AFLP markers. In this study four and eleven loci were found that were associated with underwater weight (indicative of tuber starch content) [19].

The candidate gene approach was appropriate for association mapping of tuber starch content because the biochemistry and molecular genetics of starch metabolism is well understood in plants including the potato. Many genes encoding enzymes and transporters functional in carbohydrate metabolism have been cloned, sequenced and functionally characterized [23, 24]. The candidate gene approach is less suitable though for the identification of diagnostic markers for tuber yield, as numerous, mostly unknown metabolic, regulatory and developmental processes have a potential influence on this highly complex trait. Examples for processes affecting tuber yield are carbon and energy supply [25–27] and tuberisation control [28–30]. Nevertheless, allelic variants of ten genes functional in carbohydrate metabolism, enzymatic discoloration or tuberisation were found to be associated with tuber yield and in some cases also with starch yield [12, 14, 17, 18].

Recently, new potato genomic resources became available, which allow a more comprehensive approach to the dissection of QTL and possibly the identification of the causal genes. The annotated genome sequence provides physical maps of the twelve potato chromosomes, to which any QTL can be anchored by means of linked or associated sequence based markers [31, 32]. This allows comparisons between QTL mapping experiments in different genetic backgrounds and integration of the results. A first 8.3 K SNP chip allows the genotyping of 8303 genome wide SNPs including the allele dosage in tetraploid potato [33–38]. Moreover, next generation sequencing technologies make SNP genotyping by sequencing possible, for example by restriction-site associated DNA sequencing (RADseq) [39, 40].

In this paper we address the question of the number and physical position of QTL for tuber yield, starch content and starch yield based on genome wide SNP genotyping of tetraploid potato cultivars using both RAD sequencing and the 8.3 K SolCAP SNP array [38]. We compare the results obtained with these two genotyping methods in the same three case-control populations, which were selected for having contrasting phenotypic means of tuber yield, starch content and starch yield. The results of the case-control studies are compared with the results of a genome wide association study (GWAS) using the SolCAP SNP chip, for tuber starch content in an independent population of cultivars. We also assess the reproducibility of diagnostic markers identified in previous association studies based on the candidate gene approach. In addition, we report novel candidate genes for tuber yield, starch content and starch yield.

## Methods

### Plant material and phenotypes

Two populations of tetraploid varieties and breeding clones were used for association mapping. The QUEST population consisted of 264 varieties and breeding cloness, which were evaluated in Northern Spain in two years at two locations for tuber yield (TY), starch content (TSC) and starch yield (TSY) [12]. A subset of 90 genotypes of the QUEST population was selected for SNP genotyping with the 8.3 k SolCAP potato SNP array and by RAD sequencing, based on the phenotype of TY, TSC and TSY (Additional file 1). The PIN184 population comprised 184 tetraploid breeding clones and is described in [41]. This population was evaluated in Northern Germany in three years at two locations for tuber starch content (TSC) measured by specific gravity [42]. Adjusted entry means for TSC, TY and TSY were calculated as described [12, 41] and are used throughout in this study.

### RAD library preparation and sequencing

Ninety six RAD (Restriction site Associated DNA) libraries for paired-end sequencing were prepared from 90 QUEST genotypes and six duplicate genotypes according to Etter et al. [43] with modifications according to Bus et al. [44]. In brief, 2 μg genomic DNA of each genotype were restricted with *Kpn*I. The 96 libraries were individually barcoded with a custom made P1 adapter, which was ligated to the *Kpn*I cut sites. Adapter ligations were incubated at 16 °C overnight. The barcodes were designed to be separated from each other by at least six mutational steps. The 96 libraries were then pooled into two samples comprising 48 genotypes each. The two samples were sheared by ultrasound and DNA fragments of 300 to 400 bp were extracted after size separation from 1.5% agarose gels. An A-overhang was added to the blunt ends and the common P2 adapter was ligated as before to the template. P1 and P2 adapters were kindly provided by Anja Bus and Benjamin Stich (MPI for Plant Breeding Research). After a PCR (polymerase chain reaction) enrichment step, a second size selection of 300–400 bp fragments was performed. Sample quality was assessed on a 2100 Bioanalyzer (Agilent Technologies, Böblingen, Germany). The two samples were custom sequenced in two lanes of an Illumina HiSeq2000 system at the Max-Planck Genome Centre Cologne using GAIIx chemistry.

### Sequence analysis and SNP detection after RAD sequencing

Paired-end reads were combined from both sequencing lanes and sorted according to the barcode, allowing maximally one mismatch. No mismatch was allowed at the restriction site. The sequences were mapped against the potato genome sequence (version v2.1.11, 32] using the Bowtie software package version 0.12.8 with default settings [45]. Reads that did not map to a unique genomic position were excluded from further analysis. The sequence reads of individual genotypes were assigned to the three case-control populations for TSC, TY and TSY. Further analysis was performed with the sequence reads pooled for cases and controls. Bi-allelic SNPs were called within case-control populations using the Genome Analysis Toolkit GATK [46]. Fisher’s exact test was implemented with a custom-made Perl script to test wether the allele frequencies of a biallelic SNP differed between a pair of genotype pools with contrasting phenotypic means. FDR (false discovery rate) values were obtained with Bonferroni’s correction for multiple testing and SNPs significant at FDR < 0.05 were selected. The SNPs in annotated loci were obtained by combining the SNP genomic positions (pseudomolcules version v2.2.11) with the annotation file of the potato genome sequence [32]. Non-synonymous SNPs were identified with the software package SnpEff [47].

### Genome wide genotyping with the SolCAP SNP array

Genomic DNA was extracted from freeze dried leaf tissue as described [12, 41]. DNA samples were diluted to 50 ng/μl AE-buffer (10 mM Tris-Cl, 0.5 mM EDTA, pH 9.0). The same 90 QUEST genotypes as used for RAD sequencing plus six technical replicates and the complete PIN184 population were custom genotyped with the Infinium 8303 potato array (8.3 k SolCAP SNP array) at the Life & Brain Center (Department of Genomics, Bonn, Germany) on an Illumina iScan system, applying the Infinium assay. The genotypes of bi-allelic SNP markers in the PIN184 population were called using the software package ‘fitTetra’ [48]. The package was run using the function fitTetra with the option try. HW = F. SNP genotypes were assigned manually to the 96 QUEST genotypes using GenomeStudio software version 2011.1 (Illumina). One of the five possible genotypes (*AAAA, AAAB, AABB, ABBB, BBBB*) was assigned to each individual for each SNP. The quality of the genotype clusters of differential/associated SNPs was visually examined a posteriori with GenomeStudio software version 2011.1 (Illumina) and very low quality SNPs were discarded. To detect SNPs with differential allele frequency in the QUEST case-control populations, Pearson’s goodness-of-fit test (chi-square test) was applied to test the null hypothesis that the allele frequencies of a bi-allelic SNP in a pair of genotype pools with contrasting phenotypic means were equal. The null hypothesis was rejected at the significance level α < 0.01. The chi-square tests were performed using SAS software (version 9.1). Duplicates of six QUEST genotypes were included in the analysis. For each genotype duplicate Pearson’s correlation coefficient between all genotypic data was calculated to assess the technical repeatability of the genotyping method.

### SNP detection by amplicon sequencing and pyrosequencing

PCR amplification of genomic fragments, amplicon sequencing, pyrosequencing, SNP detection and scoring were performed as described previously [12, 49]. Amplicon sequences, primer sequences and annealing temperatures are provided in Additional file 2.

### Association analysis

In the QUEST population, association analysis of SNPs in candidate genes was performed as described in Schönhals et al. [12] using a mixed linear model which included kinship and population structure. Kinship and population structure were calculated based on the marker data of 183 microsatellite alleles at 29 loci distributed on the twelve potato chromosomes. In the PIN184 population, genome wide association analysis of SolCAP SNPs was performed using the kinship model K1 (a mixed linear model) described in Mosquera et al. [36]. The kinship matrix was calculated based on the genotypes of 241 SolCAP SNPs that were selected for equal distribution on the twelve potato chromosomes.

## Results

### Case-control populations for tuber starch content (TSC), yield (TY) and starch yield (TSY)

**Fig. 1:**
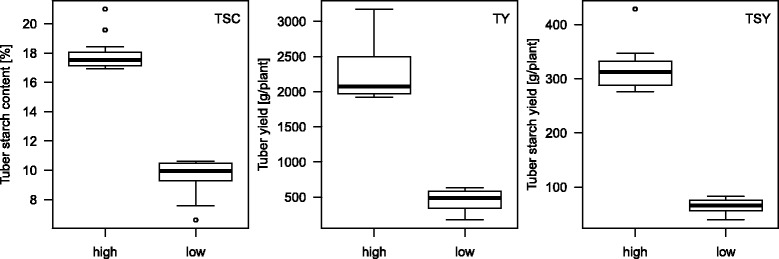
Box plots of the QUEST case-control populations for TSC, TY and TSY

Based on the adjusted means for TSC, TY and TSY of 264 genotypes of the QUEST population [12], ninety genotypes were selected in order to assemble genotype groups with most contrasting phenotypic means (case-control populations). The same genotype could be member of more than one group (Fig. 1, Additional file 1). Each of the case-control populations for TSC and TY consisted of two groups of 24 genotypes with high (cases) and low (controls) mean values of TSC and TY. The case-control population for TSY consisted of 21 genotypes with high TSY (cases) and 24 genotypes with low TSY (controls). The mean phenotypic differences between the HIGH and LOW groups were highly significant (*p* < 0.001). The two groups selected for TSC differed also for TSY (*p* < 0.001) but not for TY (*p* = 0.732), the groups selected for TY differed also for TSY (*p* < 0.001) but not for TSC (*p* = 0.576), whereas the groups selected for TSY differed also for TSC and TY (*p* < 0.001).

### SNPs with differential allele frequencies in QUEST case-control populations obtained by RAD sequencing (RADseq)

**Fig. 2:**
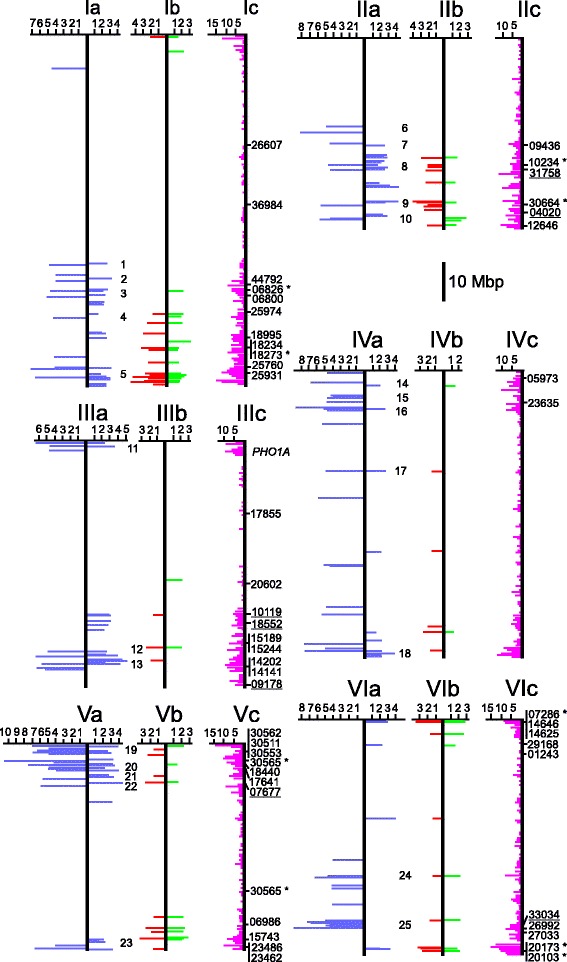
Physical maps of potato chromosomes I to VI. The maps are based on the pseudomolecules version 4.03 [31], which are represented as vertical bars. Chromosomes **a:** to the left are shown as horizontal blue lines the positions of the genes detected by association of SolCAP SNPs with TSC in the PIN184 population (details in Additional file 9). To the right are shown as horizontal blue lines the positions of the genes tagged by SolCAP SNPs with differential allele frequency in the QUEST case-control population for TSC (details in Additional file 8). The length of the lines is proportional to the *p* value. The scale (− log10(p)) is shown on top of the chromosome maps. Chromosomes **b**: To the left are shown as horizontal red lines the positions of the genes tagged by SolCAP SNPs with differential allele frequency in the QUEST case-control population for TY. On the right are shown as horizontal green lines the positions of the genes tagged by SolCAP SNPs with differential allele frequency in the QUEST case-control population for TSY; otherwise as in chromosomes **a** (details in Additional file 8). The numbers between chromosomes **a** and **b** indicate the positions of the 46 genomic regions, where QTL for TSC detected by GWAS in the PIN184 population overlap with QTL for TSC, TY and TSY detected by differential SNPs in the QUEST case-control populations (details in Additional files 4 and 5). Chromosomes **c**: To the left is shown as horizontal purple lines the frequency distribution of 3144 genes with differential RADseq SNPs, at least one with FDR < 0.001 (Table 1), in the QUEST case-control populations. The scale (No. of genes per 0.5 Mbp) is shown on top of the chromosome maps (details in Additional files 4 and 5). To the right are shown the positions of 71 genes with RADseq SNPs differential in all three case-control populations and with unidirectional effect on TSC, TY and TSY (Table 2). They are identified by the last five digits of the PGSC0003DMG locus number. Included are here also the positions of the eleven candidate genes with differential SNPs that were tested for association in the whole QUEST population (indicated by *) (Table 3), and the positions of 14 candidate genes (underlined) associated with TSC, TY or TSY in previous association studies that were also detected in the QUEST case-control populations (Table 6). The approximate position of *PHO1A* on the short arm of chromosome III, which is not included in pseudomolecules version 4.03, is also shown

**Fig. 3:**
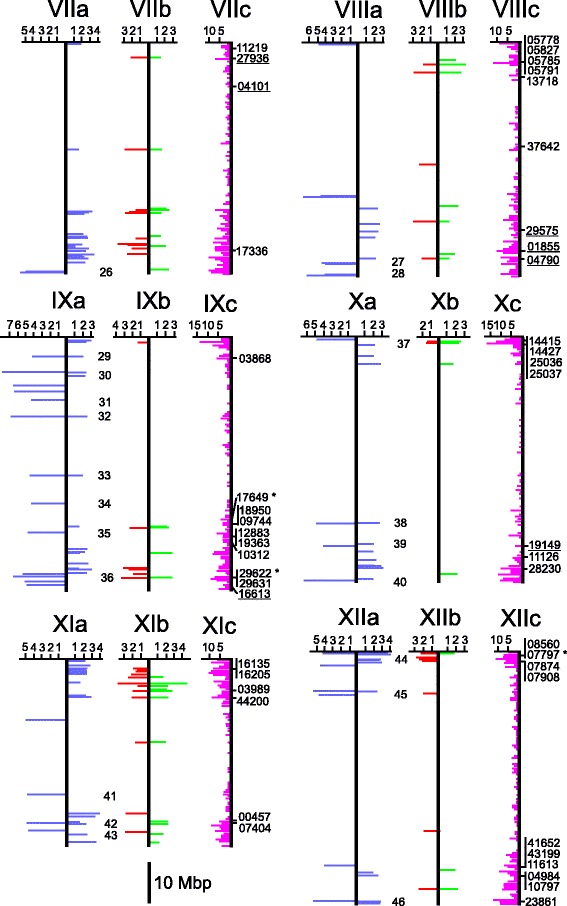
Physical maps of potato chromosomes VII to XII. See Fig. 2 for details

The case-control populations for TSC, TY and TSY were genotyped by RAD sequencing (European Nucleotide Archive (ENA): Study accession No. PRJEB10900, http://www.ebi.ac.uk/ena/data/view/PRJEB10900). SNPs were identified by mapping the sequence reads to the potato genome sequence. SNP allele frequencies were estimated after pooling the data of the HIGH and LOW groups for TSC, TY and TSY. The results are summerized in Table 1. Of approximately 600,000 SNPs detected in each case-control population, 10.2%, 7.7% and 3.4% showed differential allele frequencies (FDR < 0.05) between the case-control populations for TSC, TY and TSY, respectively (Table 1: data filtering criteria 1 and 2). Further analysis was restricted to 25,501 SNPs with differential allele frequencies (FDR < 0.05) in 6664 annotated genes (Table 1: data filtering criteria 3 and 4, Additional file 3). Eighty percent of the annotated genes (5333) contained two or more differential SNPs, predominantly for TSC and TY, and 34% of these genes (1799) contained multiple SNPs differential for TSC, TY and TSY singularly and combinations thereof (see below). Eighteen percent of the differential SNPs (4668, FDR < 0.05) caused amino acid changes in 2308 deduced proteins (Table 1: data filtering criteria 5 and 6). Stringent filtering of the data for differential SNPs with FDR < 0.0001 and causing amino acid changes resulted in 582 SNPs in 450 genes (Table 1, data filtering criterion 7, genes are highlighted green in Additional file 4). One or more differential SNPs with FDR < 0.001 were present in 3144 genes (Table 1: data filtering criterion 8, Additional file 4). The distribution of the positions of the corresponding loci on the physical chromosome maps (Additional file 5) showed that the majority of 0.5 Mbp intervals contained none, one or two genes with differential SNPs, particularly in the central, gene poor regions of the chromosomes. The number of genes per 0.5 Mbp increased toward distal, gene rich chromosomal regions. On top of this general distribution of genes in the potato genome [32], 449 0.5 Mbp intevals with 3 to 17 genes were observed (31% of all 0.5 Mbp intervals), which were fused into 204 peaks between 0.5 and 4 Mbp wide (Additional file 5). The frequency distribution of the 3144 loci on the physical maps of the twelve potato chromosomes at the resolution of 0.5 Mbp is shown in Figs. 2 and 3 (chromosomes c). With few exceptions, where peaks included differential SNPs only for TSC and TY, the peaks included differential SNPs for all three traits. The 27 most prominent peaks included between 10 and 17 genes per 0.5 Mbp (highlighted yellow in Additional file 5).

**Table 1:** Summary statistics of differential SNPs and their corresponding genes identified by RAD sequencing

Data filtering criteria	Total No.	TSC	TY	TSY	TSC and TY	TSC and TSY	TY and TSY	TSC, TY and TSY
1. Total No. of SNPs	nd	579,352	601,837	598,703	nd	nd	nd	nd
2. No. of SNPs with differential allele frequency (FDR < 0.05)	nd	58,850	46,542	20,402	nd	nd	nd	nd
3. No. of SNPs with differential allele frequency in annotated genes (FDR < 0.05)	25,501	10,986	5660	2207	3005	665	2330	648
4. No. of annotated genes with differential SNPs (FDR < 0.05)	6664	1456	654	218	1575	430	532	1799
5. No. of SNPs with differential allele frequency in annotated genes causing amino acid changes (FDR < 0.05)	4668	1993	1043	403	537	135	433	124
6. No. of annotated genes with differential SNPs causing amino acid changes (FDR < 0.05)	2308	378	146	43	622	162	168	789
7. No. of SNPs with differential allele frequency in annotated genes causing amino acid changes (FDR < 0.0001)	582	310	148	58	40	1	23	2
8. No. of annotated genes with at least one differential SNP with FDR < 0.001	3144	431	81	13	842	194	257	1326

*nd* not determined

Twenty three percent (6000) of the SNPs had differential allele frequencies in two case-control populations, in TSC and TY (3005), TSC and TSY (665) or in TY and TSY (2330) (FDR < 0.05) (Table 1: data filtering criterion 3, Additional file 6). The 3005 SNPs with differential allele frequencies in both the TSC and TY case-control populations showed antagonistic effects: the allele frequency in the HIGH-TSC and LOW-TY population was higher compared with the LOW-TSC and HIGH-TY population, and vice versa. This indicated that the allele with a positive effect on TSC was compromised by a negative effect on TY and vice versa. SNPs with differential allele frequencies in both TSC and TSY, and both TY and TSY case-control populations showed the same direction of effect. This was to be expected as TSY is derived from TSC and TY. Six hundred and forty eight SNPs in 468 genes showed differential allele frequencies in all three case-control populations (Additional file 7). In the majority of these cases, a positive effect of the SNP allele on TY and TSY was also confounded by a negative effect on TSC and vice versa. Importantly, the alleles of 88 SNPs in 71 genes showed the same or a neutral direction of effect on all three traits (highlighted green in Additional file 7). The positions of these 71 genes on the physical chromosome maps are shown in Figs. 2 and 3 (chromosomes c). Twenty six of the 88 SNPs in 18 genes caused amino acid changes (Table 2).

**Table 2:** Differential RADseq SNPs causing amino acid changes with unidirectional allele effect on TSC, TY and TSY in QUEST case-control populations

SNP position (v2.1.11)	Amino acid change	Locus PGSC0003	Locus position [Mbp] (v4.03)	Annotation	Function category
chr01:37,823,297	G/R	DMG400036984	chr01: 42.92	Gene of unknown function	Unknown
chr01:63,201,542	M/K	DMG400025974	chr01: 70.21	EF hand family protein	Calcium binding (IPR002048)^a^
chr01:63,201,571	K/N	DMG400025974	chr01: 70.21	EF hand family protein	dito
chr01:63,201,582	V/F	DMG400025974	chr01: 70.21	EF hand family protein	dito
chr01:76,804,110	G/R	DMG400025760	chr01: 83.89	Homeobox protein HAT3.1	Transcription regulation (uniprot/Q04996)^b^
chr01:78,272,215	H/Y	DMG400025931	chr01: 85.36	GTP-binding protein alpha subunit, gna	Signalling [59]
chr01:78,272,224	Y/D	DMG400025931	chr01: 85.36	GTP-binding protein alpha subunit, gna	dito
chr02:46,113,238	R/G	DMG400012646	chr02: 47.66	Conserved gene of unknown function	Unknown
chr05:4,555,061	D/G	DMG400018440	chr05: 4.78	Conserved gene of unknown function	Unknown
chr05:5,636,330	S/F	DMG400017641	chr05: 5.99	Conserved gene of unknown function	Unknown
chr06:47,805,636	C/G	DMG402027033	chr06: 53.48	Zinc finger family protein	Multiple regulatory functions [60]
chr07:872,052	I/L	DMG400011219	chr07: 1.53	Tetratricopeptide repeat protein	Protein-protein interactions, complex assembly (IPR013026)
chr07:872,053	I/N	DMG400011219	chr07: 1.53	Tetratricopeptide repeat protein	dito
chr08:5,319,820	L/F	DMG400005778	chr08: 4.84	NADPH-dependent thioredoxin reductase B	Defense to stress [61]
chr10:1,920,325	A/S	DMG400025036	chr10: 1.91	Nucleic acid binding protein	Unknown
chr11:3,818,033	T/I	DMG400016135	chr11: 2.31	Tyrosine phosphatase	Posttranlational regulation [62]
chr11:9,422,323	V/L	DMG400044200	chr11: 9.48	Gene of unknown function	Unknown
chr11:36,440,960	I/L	DMG400000457	chr11: 39.25	Lethal leaf spot 1	Chlorophyll catabolism, cell death [63]
chr11:36,440,962	S/N	DMG400000457	chr11: 39.25	Lethal leaf spot 1	dito
chr12:50,607,969	M/V	DMG400041652	chr12: 52.23	Microsomal oleic acid desaturase	Lipid metabolism
chr12:50,608,022	Q/H	DMG400041652	chr12: 52.23	Microsomal oleic acid desaturase	dito
chr12:50,526,275	D/N	DMG400011613	chr12: 52.32	ABC transporter family protein	Transport [64]
chr12:51,537,813	E/G	DMG400004984	chr12: 53.87	Conserved gene of unknown function	Unknown
chr12:58,658,881	P/S	DMG400023861	chr12: 60.72	Extensin Ext1	Cell wall [57]
chr12:58,658,917	E/K	DMG400023861	chr12: 60.72	Extensin Ext1	dito
chr12:58,658,927	S/L	DMG400023861	chr12: 60.72	Extensin Ext1	dito

^a^Entry in the InterPro database (http://www.ebi.ac.uk/interpro/)

^b^Entry in the uniprot database (http://www.uniprot.org/)

Based on the annotation, the function of 25% of all genes with differential SNPs was unknown. Besides that, the most frequent functional categories represented by multiple genes with differential SNPs were: signal perception and transduction (receptors, protein kinases and phosphatases, calmodulin binding proteins), transcriptional regulation (transcription factors), protein synthesis (e.g. ribosomal proteins, translation initiation factors), protein degradation by the 26S proteasome pathway (e.g. F-box proteins, ubiquitin metabolising genes) or by proteases, chaperones (heat shock proteins, DnaJ), organelle biogenesis and function (Pentatricopeptide repeat containing proteins), transport (e.g. amino acids, sugars, nitrate, peptides), cell wall synthesis and modification (e.g. extensins, expansins, cellulose synthases), chromatin organization and modification (e.g. ‘chromo’ domain containing proteins), biotic stress (nucleotide binding site – leucine rich repeat (NBS-LRR) type resistance genes) and retrotransposons (gag-pol polyproteins, integrase core domain containing proteins) (Additional files 3, 4, 5, 6 and 7).

### SolCAP SNPs with differential allele frequencies in QUEST case-control populations

The same genotypes as used for genotyping by RAD sequencing were genotyped for 8303 SolCAP SNPs. Genotyping was highly reproducible as assessed by comparing the results of replicated genotypes. Genotype calling resulted in 7454 SNPs (89.8%) that could be assigned to one of the five genotype classes *AAAA, AAAB, AABB, ABBB* and *BBBB*. The SNPs were tested for differential allele frequency between cases and controls and ordered according to the *p*-value. SNPs with differential allele frequencies at *p* < 0.01 were selected and further analysed. This resulted in 204 differential SNPs in the TSC, 85 in the TY and 55 in the TSY case-control populations. Eight of these SNPs did not map to the physical chromosome maps and were not considered further. When combining the differential SNPs from the three case-control populations, differential allele frequencies of total 306 SNPs in 275 genes on all potato chromosomes were observed (Figs. 2 and 3, chromosomes a and b, Additional file 8). Eighty three percent of the differential SolCAP SNPs co-localized within less than 0.5 Mbp with 81 peaks in the distribution of genes with differential RADseq SNPs (Additional file 8). If a SNP was significant at *p* < 0.01 for one trait, values of *p* < 0.05 for the other two traits were also included in the comparison among the case-control populations. Based on this comparison, forty five SNPs showed differential allele frequencies in both the TSC and TY case-control populations. As observed for the same category of SNPs in RADseq, these SNPs showed an antagonistic allele effect: the allele with higher frequency in the HIGH-TSC compared to the LOW-TSC population had lower frequency in the HIGH-TY compared to the LOW-TY population and vice versa. Forty three and seventeen SNPs showed differential allele frequencies in both the TY and TSY and both the TSC and TSY case-control populations, respectively. The allele effect on both traits had the same direction (Additional file 8). Fifteen SNPs showed differential allele frequencies in all three case-control populations. With one exception (solcap_snp_c1_5656), the allele effect in the TSC population was opposite to the effect in both the TY and TSY populations (Additional file 8). Solcap_snp_c1_5656 was located in locus DMG401025958 annotated as ‘glycine-rich protein’ [50]. The same gene was detected by differential RADseq SNPs.

### Association analysis of genes with differential SNPs in the QUEST case-control populations

In order to test, to what extent SNPs with differential allele frequency in the QUEST case-control populations show association with TSC, TY and/or TSY in the whole QUEST population (*n* = 264), we selected eleven candidate genes for association analysis (Table 3). Primers for PCR amplification were designed for gene fragments which included 32 differential SNPs in the case-control populations. In total 83 SNPs and one deletion were scored in the whole QUEST population, either in amplicon sequences or by pyrosequencing (Additional file 2). Based on association analysis using a mixed linear model which considered kinship and population structure, twenty SNPs in nine genes were associated with TSC, TY, TSY or all three traits (Table 4). Sixteen of these twenty associated SNPs were also detected by differential allele frequencies in the case-control populations, whereas four were novel.

**Table 3:** The genes analysed for association with TSC, TY and TSY in the whole QUEST population

Locus^a^	Chromosome: [Mbp] (4.03)	Annotation (locus acronym)	Differential SNPs (No. of SNPs in RADseq)	Differential for traits (No. of SNPs)	Function category
DMG401006826	I: 64.5	Aldehyde oxidase (*AOX*)	RADseq (3 SNPs *)^b^	TY (3), TSY (3)	Hormone synthesis, reactive oxygen species [65]
DMG400018273	I: 79.2	NADPH:adrenodoxin oxidoreductase, mitochondrial (*ADXR*)	RADseq (8 SNPs)	TSC (1), TY (7), TSY (5)	Steroid metabolism (uniprot/P22570)^c^
DMG400010234	II: 32.0	Cysteine protease Cp5 (*CP5*)	RADseq (13 SNPs)	TSC (13), TY (8)	Protein degradation [66]
DMG400030664	II: 42.1	Sporulation protein RMD5 (*SpRMD5*)	RADseq (25 SNPs)	TSC (8), TY (19), TSY (14)	Regulation of gluconeogenesis (uniprot/Q12508)
DMG400030565	V: 3.7	Fructose-bisphosphate aldolase *(FBA)*	solcap_snp_c2_11924	TSC	Glycolysis, gluconeogenesis, Calvin cycle [67]
DMG400007286	VI: 0.2	Chloroplast protein 12 (*CP12.1*)	solcap_snp_c2_54011	TY, TSY, TSC	Calvin cycle [68]
DMG400020173	VI: 58.2	Mak, serine/threonine protein kinase (*MAK*)	RADseq (9 SNPs *)	TSC (4), TY (9), TSY (6)	Posttranslational regulation https://www.ncbi.nlm.nih.gov/gene/4117
DMG400020103	VI: 58.2	Glycosyltransferase QUASIMODO1 (*QUA1)*	solcap_snp_c2_9201, 9202, 9203	TY (3), TSY (2)	Pectin synthesis, cell wall [69]
DMG400017649	IX: 44.1	Pentatricopeptide repeat-containing protein (*PPR*)	RADseq (13 SNPs *)	TSC (11), TY (11)	Organelle biogenesis and function [70, 71]
DMG400029622	IX: 58.4	60S acidic ribosomal protein P0 (*RP60S*)	solcap_snp_c2_3063	TY, TSY	Protein synthesis [72, 73]
DMG400007797	XII: 1.3	Citrate synthase (*CIS*)	RADseq (3 SNPs *), solcap_snp_c2_25372	TSC (2), TY (2)	Tricarboxylic acid cycle, carbohydrate metabolism (uniprot/Q43175)

^a^Details in Additional files 3 and 8

^b^* indicates that at least one SNP caused an amino acid change

^c^Entry in the uniprot database (http://www.uniprot.org/)

**Table 4:** SNPs associated with TSC, TY and TSY in the QUEST population (*n* = 264)

SNP name	SNP alleles *phu/tbr*	Chromosome	Minor allele frequency^a^	TSC (R^2^)^b^	TY (R^2^)^b^	TSY (R^2^)^b^	SNP differential in case-control population for
*ADXR_SNP5705 (5710)* ^c^	T/C	I	0.491 (C)	ns	* (2.0) ↑	** (3.5) ↑	TY, TSY, TSC
*CP5_SNP3041*	C/T	II	0.442 (C)	*** (5.1) ↑	ns	ns	TSC, TY
*FBA_SNP_c2_11924*	C/T	V	0.103 (T)	** (3.0) ↑	* (1.4) ↑	** (3.8) ↑	TSC
*CP12.1_SNP_c2_54011*	C/T	VI	0.082 (T)	ns	** (3.1) ↓	* (2.1) ↓	TY, TSY, TSC
*MAK_SNP7832 (7475, 7634 7588)*	T/C	VI	0.467 (T)	ns	*** (5.0) ↓	** (3.5) ↓	TY, TSY
*MAK_SNP7576*	G/A	VI	0.491 (A)	ns	*** (4.6) ↓	*** (5.9) ↓	TSC, TY, TSY
*MAK_SNP7884*	A/C	VI	0.113 (A)	ns	** (2.8) ↑	* (1.5) ↑	novel
*QUA1_SNP_c2_9204*	G/A	VI	0.452 (G)	ns	** (2.7) ↓	* (1.8) -	novel
*QUA1_SNP9502*	G/A	VI	0.070 (G)	ns	** (3.3) ↑	* (1.8) -	novel
*QUA1_SNP_c2_9203*	C/G	VI	0.453 (C)	ns	** (3.0) ↓	* (2.2) -	TY, TSY
*PPR_SNP7181*	T/C	IX	0.495 (C)	*** (4.8) ↑	ns	ns	TSC, TY
*PPR_SNP7037 (7077)*	C/T	IX	0.440 (T)	** (3.1) ↑	ns	ns	TSC, TY
*PPR_SNP7083*	A/G	IX	0.318 (G)	** (2.2) -	ns	ns	TSC, TY
*RP60S_SNP_c2_3063*	T/C	IX	0.157 (T)	* (1.3) ↑	* (2.0) ↑	** (3.9) ↑	TY, TSY
*CIS_SNP4479*	C/G	XII	0.438 (C)	*** (4.2) ↓	ns	ns	novel

Associations of SNPs with a minimum minor allele frequency of 1% and at least one association at *p* < 0.01 are shown

^a^The minor frequency allele is shown in parenthesis

^b^ns = not significant at α =0.05; * significant at α = 0.05, ** significant at α = 0.01, *** significant at α = 0.001; Arrows indicate the effect of the minor frequency SNP allele on the trait compared to the population mean: ↑ increasing, ↓ decreasing, − ambiguous

^c^Numbers in parenthesis identify SNPs in nearly complete LD that showed similar associations

### Association analysis of SolCAP SNPs with tuber starch content in the PIN184 population

**Fig. 4:**
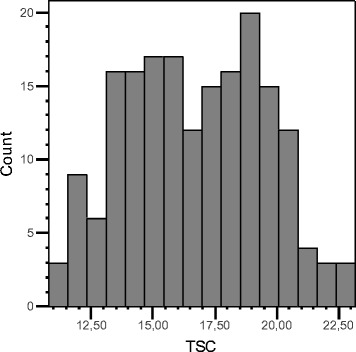
Histogram of adjusted means of tuber starch content (TSC) in the PIN184 population

The PIN184 population was evaluated for tuber starch content over three years in replicated field trials. The phenotypic distribution of TSC (adjusted means) in the PIN184 population is shown in Fig. 4. Genotyping the PIN184 population for 8303 SolCAP SNPs yielded 6286 SNPs with genotype calls [36]. Association analysis using a mixed model that corrected for kinship resulted in 127 SolCAP SNPs in 117 genes which were associated with TSC at *p* < 10^−4^. Seventy five percent of the associated SolCAP SNPs co-localized within less than 0.5 Mbp with 50 peaks in the frequency distribution of genes with differential RADseq SNPs in the QUEST case-control populations (Additional file 9). The positions of the genes with associated SolCAP SNPs on the potato physical chromosome maps are shown in Figs. 2 and 3, chromosomes a.

### Identity between SNPs, genes and genomic regions identified by different genotyping methods and in different genetic backgrounds

Identity was low between differential or associated SNPs detected in the three experiments (SolCAP SNP genotyping and RAD sequencing in the QUEST case-control populations, SolCAP SNP genotyping in the PIN184 poplation). Six of 306 SolCAP SNPs with differential allele frequency in the QUEST case-control populations were also detected by RAD sequencing in the same populations (SolCAP SNPs c2_14491, c2_16424, c2_2506, c2_7558, c2_22939, c2_12265, Additional file 8). The six corresponding genes were annotated as NBS-LRR protein (DMG400006800), zeta-carotene desaturase (DMG400022473), xyloglucan endotransglucosylase/hydrolase 1 (DMG400024755), mannose-6-phosphate isomerase (DMG400026392), serine-threonine protein kinase (DMG400043061) and CSN5 protein (DMG400009240). Seven of 127 SolCAP SNPs associated with TSC in the PIN184 population had differential allele frequencies in one, two or all three QUEST case-control populations. Five were detected by SolCAP SNP genotyping (c2_13751, c2_4708, c2_21313, c2_26858, c2_50302) and two by RAD sequencing (c2_11766, c2_1918) (Additional file 9). The corresponding genes encoded a conserved gene of unknown function (DMG400006766), phenylpropanoid: glucosyltransferase 1 (DMG400025878), starch phosphorylase PHO1a (not annotated), transcription factor B3 (DMG400027236), a methyltransferase (DMG400031262, see below), a serine-threonine protein kinase (DMG400000810) and a kinase (DMG400029885, see below).

Higher correspondence was observed between the experiments when the comparison was based on the genes containing differential or associated SNPs. Seventy two identical genes were detected by SolCAP SNP genotyping as well as RAD sequencing in one, two or all three QUEST case-control populations (Additional file 10). Importantly, forty two identical genes were detected in both the PIN184 and the QUEST case-control populations. Three genes, annotated as methyltransferase (DMG400031262), kinase (DMG400029885) and copper ion binding protein (DMG400027986) on chromosomes V and IX were detected in all three experiments (Table 5). When the window for the comparison was widended to genomic regions, where genes associated with TSC in the PIN184 population co-localized with differential SNPs for TSC, TY and TSY in the QUEST case-control populations, either within 0.5 Mbp with SolCAP SNPs and/or with peaks in the frequency distribution of genes with differential RADseq SNPs, 46 genomic regions between one and four Mbp width were distinguished (Figs. 2 and 3, Additional files 4, 8 and 9).

**Table 5:** Identical genes detected by two genome wide genotyping methods in the QUEST case-control and PIN184 populations

Locus PGSC0003	Chromosome: Mbp (v4.03)	Annotation	QUEST cases/controls SolCAP SNPs	QUEST cases/controls RADseq SNPs	PIN184 GWAS SolCAP SNPs	Function category
DMG400006283	I:60.5	ABC-transporter	-	TSC	TSC	Transport [64]
DMG400006766	I:64.6	Conserved gene of unknown function	TSC, TSY	-	TSC	Unknown
DMG400000010	I:71.4	Glycosyltransferase	-	TY	TSC	Cell wall biosynthesis [74]
DMG400025878	I:84.2	Phenylpropanoid:glycosyltransferase 1	TSC	-	TSC	Phenylpropanoid metabolism [75]
DMG400030954	I:86.4	Beta-galactosidase	-	TSC, TY, TSY	TSC	Carbohydrate metabolism (uniprot/P48980)
DMG400003324	II:22.6	Squalene epoxidase	-	TY, TSY	TSC	Cyclic triterpenoid biosynthesis [76]
DMG400006975	II:26.7	Chloride channel/carrier, CLC-Nt2	-	TSC, TY, TSY	TSC	Transport [77]
DMG400001369	II:45.4	Kinase	-	TSC, TY, TSY	TSC	Phosphorylation of specific substrates (proteins, lipids, carbohydrates and others)
Solyc03g065340 ^3^	III:?	Starch phosphorylase PHO1a	TSC	-	TSC	Starch degradation [17]
DMG400025328	III:53.3	Plasma membrane ATPase 3	-	TY, TSY	TSC	Providing energy for transport [78]
DMG400024596	III:54.1	Protein kinase ATMRK1	-	TSC	TSC	Posttranslational regulation [79]
DMG401024510	III:54.6	Enolase	TSC	-	TSC	Glycolysis, central metabolism
DMG400014223	III:57.6	4-coumarate-CoA ligase 2	-	TSC, TY	TSC	Phenylpropanoid biosynthesis (uniprot/O24146)
DMG400029505	IV:3.0	NB-LRR protein required for HR- associated cell death 1 (NRC1)	-	TSC, TY	TSC	Biotic stress [80]
DMG400011479	IV:6.2	Glycogenin-like starch initiation protein 6	-	TSC, TY	TSC	Carbohydrate metabolism (AT5G18480)
DMG400027236	IV:9.8	Transcriptional factor B3	TSC	-	TSC	Transcription regulation (IPR003340)
DMG400003751	IV:70.2	Bel1 homeotic protein	-	TSC, TY, TSY	TSC	Development [81]
DMG400028325	V:0.32	Heparanase, family-79 endo-β-glucuronidase	-	TSC, TY, TSY	TSC	Carbohydrate metabolism https://www.cazypedia.org/index.php/Glycoside_Hydrolase_Family_79
Solyc05g010320 ^3^	V:1.4	Chalcone-flavonone isomerase	TSC	-	TSC	Anthocyanin biosynthesis [82]
DMG400000810	V:2.0	Di-glucose binding protein with Leucine-rich repeat domain, serine-threonine protein kinase	-	TSC, TY, TSY	TSC	Posttranslational regulation [79]
DMG401012961	V:2.1	Aspartic proteinase nepenthesin-1	-	TY, TSY	TSC	Protein degradation [83]
DMG400031262	V:5.0	Methyltransferase	TSC, TSY	TSC, TY, TSY	TSC	Methylation of specific substrates
Solyc05g007070 ^3^	V:5.9	Alpha amylase 2	TSC	-	TSC	Starch degradation
Solyc05g007030 ^3^	V:6.0	AT3g17900/MEB5_12	TSC	-	TSC	Unknown
DMG400018605	V:10.3	Binding protein	-	TY, TSY	TSC	Unknown
DMG400023508	V:50.6	Kinase	-	TSC, TY, TSY	TSC	Phosphorylation of specific substrates (proteins, lipids, carbohydrates and others)
DMG400004062	VI:51.2	DOF domain class transcription factor	-	TSC, TSY	TSC	Transcription regulation [84]
DMG400033051	VI:51.5	Conserved gene of unknown function	-	TSC	TSC	Unknown
DMG400033034	VI:51.9	Plasma membrane H^+^-ATPase	-	TSC, TY, TSY	TSC	Providing energy for transport [78]
DMG402026985	VI:52.6	Kinase	-	TSC	TSC	Phosphorylation of specific substrates (proteins, lipids, carbohydrates and others)
DMG400012129	VIII:56.4	Beta-amylase 8	-	TSC, TY	TSC	Starch degradation
DMG400003874	IX: 4.8	Splicing factor 3A subunit	-	TY, TSY	TSC	mRNA processing
DMG400023084	IX:8.5	LMBR1 integral membrane family protein	-	TSC	TSC	Lysosomal membrane
DMG400004415	IX:15.6	Kinase	-	TSC	TSC	Phosphorylation of specific substrates (proteins, lipids, carbohydrates and others)
DMG400029885	IX:19.2	Kinase	TSC	TSC, TY	TSC	Phosphorylation of specific substrates (proteins, lipids, carbohydrates and others)
DMG400027986	IX: 33.7	Copper ion binding protein	TSC	TSC, TY	TSC	Copper ion transport (IPR000428)
DMG402017675	IX:40.4	Binding protein	-	TSC, TY	TSC	Unknown
DMG400019363	IX:47.7	Ribosome-recycling factor, chloroplastic	-	TSC, TY, TSY	TSC	Chloroplast biogenesis [85]
DMG400026430	IX:59.3	Beta-galactosidase	-	TSC, TY	TSC	Cell wall metabolism [86]
DMG400019131	X:50.6	Tubulin beta-1 chain	-	TSC, TY, TSY	TSC	Cytoskeleton structure (uniprot/P12411)
DMG403008663	XI:32.9	L-lactate dehydrogenase	-	TSC, TY	TSC	Anaeorbic glycolysis (IPR011304)
DMG400004642	XII:60.3	Lipoyl synthase, chloroplastic	-	TSC	TSC	Protein modification by lipoylation (uniprot/Q8LEE8)

^1^Entry in the InterPro database (http://www.ebi.ac.uk/interpro/)

^2^Entry in the uniprot database (http://www.uniprot.org/)

^3^The gene is not annotated in the potato genome. Solyc**g****** corresponds to the annotated orthologous gene in the tomato genome (https://solgenomics.net/)

## Discussion

### SNPs with differential allele frequencies in QUEST case-control populations for TSC, TY and TSY and their corresponding genes

Comparative RAD sequencing of the QUEST case-control populations resulted in a large number of differential SNPs distributed over the whole potato genome, despite a stringent Bonferroni correction for multiple testing. The genotypes in the case-control subpopulations were pooled in order to homogenize different genetic backgrounds of the individual genotypes analogous to the well known bulked segregant analysis (BSA) [51]. As in BSA, the homogenization of genetic background by genotype pooling was probably incomplete and resulted in an unknown fraction of differential SNPs that are unlinked to QTL for TSC and TY. This could be the reason for a background noise of genome wide distributed differential SNPs. The fact that only nine of eleven tested genes with differential SNPs could be validated by association analysis in the whole QUEST population also indicated a contamination of the RADseq data with false positive differential SNPs. Validation of physical linkage or eventually identity with QTL for TSC, TY and TSY of individual candidate genes is therefore required. This can be done by association analysis in the unselected QUEST population, by testing for reproducibility with different genotyping methods and in different populations as performed in this study, and by functional analysis of most promising candidate genes.

The majority of differential SNPs in the QUEST case-control populations showed differential allele frequency between the HIGH-TSC and LOW-TSC populations, based on SNP genotyping by RAD sequencing as well as by the SolCAP SNP chip. The same observation applied to the number of genes with differential SNPs. Possible reasons for this observation are that more genes control tuber starch content than tuber yield, or that many genes with small effect on tuber yield were below detection level. Alternatively, the phenotypic selection of the HIGH-TY and LOW-TY genoptypes might have been less effective due to the lower heritability of tuber yield compared with starch content [12], resulting in less power to detect genetic effects on tuber yield. Twenty percent of the SNPs differential for TSC were also differential for TY but with antagonistic allele effect. The allele with a positive effect on TSC was compromised by a negative effect on TY and vice versa. This antagonism neutralized the effects on TSY. The number of differential SNPs for TSY was therefore lower compared with its components TSC and TY. The same effect was observed for most SNPs differential in all three case-control populations. In these cases however, the allele effect on TSY was not completely neutralized by the antagonistic effect on TSC and TY. This shows that the relative amounts of starch and water, which are the major constituents of total tuber yield, are partly controlled by a common set of genes and explains the previously observed negative phenotypic correlation between tuber starch content and yield [16, 18]. In contrast, the alleles of 88 RADseq and one SolCAP SNP showed the same or a neutral direction of effect on TSC, TY and TSY. The corresponding genes were located on all chromosomes, with clusters on the south arms of chromosomes I, II, III, VI, IX and XII and the north arms of chromosomes V, VI, VIII, X, XI and XII (Figs. 2 and 3). These SNPs with unidirectional effect are particularly interesting for breeding applications, as they allow the simultaneous selection of positive alleles for tuber starch content and yield.

More SNPs were differential for both TY and TSY than for TSC and TSY, and the same was true for the corresponding genes. This indicates that tuber starch yield was more strongly influenced by genes controlling tuber yield than starch content.

Eighty percent of the genes detected by RAD sequencing contained multiple differential SNPs, and the largest fraction of these contained different SNPs that were differential for TSC, TY, TSY and combinations thereof (Table 1). Many of these SNPs were most likely redundant due to high linkage disequilibrium (LD) between SNPs within the same gene or between physically closely linked genes. A direct estimate of LD was not possible in the QUEST case-control populations though due to the necessity to pool the genotypes. The allele counts of individual genotypes were insufficient in numbers for statistical analysis. Symptoms for the presence of LD were different SNPs with nearly identical allele counts in the HIGH and LOW populations, and the local peaks observed in the frequency distribution of genes with differential RADseq SNPs on the physical chromosome maps (see below). On the other hand, the allele counts of many differential SNPs within the same locus were not obviously correlated. Genome wide genotyping of the QUEST case-control populations clearly showed that hundreds of genes influence TSC and TY. Even in case they function independently, many of them will be located physically close enough to each other such that their allele effects overlap due to LD between physically linked loci. Consequently, multiple differential SNPs with independent allele counts in the the HIGH and LOW populations will be observed within the same locus. The same observation can be explained by pleiotropic effects of multiple alleles of the same gene.

### Comparison between genome wide genotyping methods

The three QUEST case-control populations for TSC, TY and TSY were genotyped for genome wide SNPs on the one hand by RAD sequencing and on the other with the 8.3 K SolCAP SNP chip. The main difference between the two genotyping methods was the genome coverage, which was two orders of magnitude higher in RAD sequencing. The average physical distance between SNPs was approximately 1.25 kbp in RAD sequencing and 100 kbp between SolCAP SNPs. The average SNP frequency in the genome of tetraploid potatoes is one SNP in every twenty to thirty base pairs [52]. Both genotyping methods were therefore far from capturing the full DNA diversity among potato cultivars. The selection of SNPs for the SolCAP chip was technically biased by the requirements for allele detection and quantification with the Infinium SNP assay (ca. 50 bp sequence surrounding the SNP should be void of other SNPs) and to some extent biologically, as a portion of the 8303 SNPs was selected from candidate genes [38]. RAD sequencing was technically biased by the frequency and distribution of the *Kpn*I restriction site used for generating the RAD libraries. The SolCAP SNPs represented a small fraction of the DNA variation among five US varieties and the European variety Bintje [34], whereas RAD sequencing captured a larger fraction of the DNA variation of ninety mostly European cultivars and breeding clones [12]. The detection of identical, differential SolCAP SNPs by RAD sequencing was therefore rather unexpected. Nevertheless, six differential SolCAP SNPs were also detected by RAD sequencing, which confirms that the corresponding genes are closely linked to a QTL. The correspondence between the two genotyping methods increased, when the comparison was based on the identity of the genes detected with both methods but mostly with different SNPs. In this case, 72 of 275 genes (26%) with differential SolCAP SNPs were also detected by differential RADseq SNPs. When the window for the comparison was further widended to physical genome segments, where differential SolCAP SNPs co-localized with peaks in the frequency distribution of genes with differential RADseq SNPs, 83% of the differential SolCAP SNPs fulfilled this condition. This points to the fact that short distance LD within genes and longer distance LD between physically linked genes (haplotype blocks) strongly influenced the numbers of the detected differential SNPs and their corresponding genes.

The numbers of genes with differential SNPs obtained with RAD sequencing were one order of magnitude higher than with the SolCAP SNP array, most likely due to inflation by extensive LD between differential SNPs in physically closely linked genes, only one of which might have a genuine effect on the phenotype. Although a direct estimate of LD was not possible in the case-control populations (see above), we assumed that LD caused the peaks observed in the frequency distribution of genes with differential RADseq SNPs on the physical chromosome maps (Figs. 2 and 3, Additional file 5). We further assumed that the peak width from less than 0.5 Mbp up to 4 Mbp was an indicator for the size of haplotype blocks in the QUEST population. Recently, an LD decay between 0.6 and 2.5 Mbp in euchromatic regions was reported in a population of 537 tetraploid potato varieties [53], which is in good agreement with most peak widths observed in this study. The observation of larger, three to four Mbp wide peaks on chromosomes I, III, VI, X and XII might be the result of overlapping haplotype blocks and lack of resolution. We conclude from these observations that several hundred rather than several thousand genes control the natural variation of tuber starch content, yield and starch yield in the QUEST population.

### Comparison between different genetic backgrounds.

GWAS in the PIN184 population with the SolCAP SNP chip allowed a comparison of QTL for TSC in different genetic backgrounds, which were evaluated in different geographical regions. The QUEST population was evaluated in northern Spain [12] and the independent PIN184 population in northern Germany [41]. The overlap between the populations was again low on the level of identity between associated SNPs in the PIN184 and differential SNPs in the QUEST case-control populations. The seven SolCAP SNPs that were indeed identical, and the corresponding genes are particularly interesting for breeding applications, as they showed reproducible effects on TSC irrespective of genetic background and environment. Among those was the starch phosphorylase gene *PHO1a* on chromosome III, a well characterized functional candidate gene that was also associated with TSC in two additional, independent populations, although with different SNPs [14, 16, 18]. There is evidence that *PHO1a* is one of the causal genes for tuber starch content QTL [17]. On the level of gene identity, 42 of 127 (33%) genes with associated SolCAP SNPs in the PIN184 population were also detected by differential SNPs in the QUEST case-control populations (Table 5). The highest overlap between QTL in the two genetic backgrounds was again observed when the comparison was extended to physical genome segments, where associated SolCAP SNPs in the PIN184 population co-localized with differential SNPs in the QUEST case-control populations. The 46 defined genomic regions cover approximately 82 Mbp of the potato genome and include QTL for TSC, TY and TSY. Several of these QTL regions also show good correspondence with TSC- and TY- QTL detected in previous linkage studies in various diploid and tetraploid mapping populations, discussed in [12], particularly on chromosomes I (QTL regions 1 to 5), II (QTL regions 6 to 10), III (QTL regions 12 and 13), V (QTL regions 19, 20 and 23), VII (QTL region 26), VIII (QTL region 27 and 28) and XII (QTL region 46) (Figs. 2 and 3). The genes in the 46 QTL regions are promising candidates for the identification of DNA markers for all three complex traits, with diagnostic power in multiple populations and environments.

Two additional populations of tetraploid varieties and breeding clones different from the QUEST and PIN184 populations studied here, have been previously phenotyped, among other traits, for tuber starch content and yield [14, 18]. The same populations have been genotyped for DNA polymorphisms in candidate genes, the majority of which function in carbohydrate metabolism and transport. The QUEST population has also been genotyped with SNPs in several candidate genes [12]. These previous association analyses identified DNA variants in 39 genes that were associated with TSC, TY and/or TSY (details in Additional file 11). Under the cut off conditions used for data analysis in the present study, fifteen of these genes (38%) were detected by differential SNPs in the QUEST case-control populations, twelve by RADseq, one by a SolCAP SNP and two by both RADseq and SolCAP SNPs. SolCAP SNPs in two of these genes were also associated with TSC in the PIN184 population (Additional file 11, Figs. 2 and 3, chromosomes c). Less stringent conditions for *p* values and analysed genomic sequences could increase the overlap between results of the candidate gene and the genome wide approach.

Eight of the ten candidate loci associated with TSC, TY or TSY in the whole QUEST population [12] were not detected by RAD sequencing in the QUEST case-control populations. Reasons could be the incomplete genome coverage by RAD sequencing, and that the case-control study design is not fully equivalent to association mapping in an unselected population. Vice versa in the present study, only one half of 32 differential SNPs in the QUEST case-control populations but nine of the eleven corresponding genes were reproducible by association analysis in the whole QUEST population. Similarly, the *PHO1a* locus was detected by the same SolCAP SNP in the QUEST case-control population for TSC as well as in the unselected PIN184 population (see above), whereas the marker for a specific *PHO1a* allele diagnostic for higher tuber starch content [16, 17] was not associated in the whole QUEST population [12].

These comparisons show that the results obtained with different study designs and genotyping strategies in different genetic backgrounds partially overlap. Such comparisons are novel in potato genome analysis and highly useful for selecting the most promising candidate genes for breeding applications as well as further functional studies.

### Novel candidate genes for TSC, TY and TSY

RAD sequencing provided the highest genome coverage of the three experiments described in this study. RAD sequencing offered therefore the best chance that some among the 6664 genes with differential RADseq SNPs are causal for TSC-, TY- and TSY-QTL. Enriched for such genes should be the 2308 genes with differential SNPs causing amino acid changes, although we cannot exclude that some amino acid changes deduced from sequence alignment are artefacts caused by the algorithm used for mapping the sequence reads [54]. Stringent filtering for differential SNPs with the smallest FDR values (FDR < 0.0001) and causing amino acid changes resulted in 450 genes, which might be considered as primary functional candidates for TSC-, TY- and TSY-QTL. Hardly any of these genes has been functionally characterized in potato. Except one hexose transporter (DMG400031832), this set did not include genes with a direct function in starch metabolism [17]. The largest fraction comprised genes with unknown or unspecified function with respect to the pathway involved (e.g. DMG400008990, DMG400028376 and DMG400038781). Next to this, signalling, transcriptional and posttranscriptional regulation seem to have important roles for TSC and TY. Multiple genes with highly differential SNPs were in this categories, which were annotated as receptor-like kinases (e.g. DMG401027271), transcription factors (e.g. DMG400000459), protein kinases and phosphatases (e.g. DMG400002885, DMG400011440), F-box proteins and ubiquitin ligases (e.g. DMG401031260, DMG400009902). In the same context of cellular regulation belongs protein homeostasis regulated by synthesis (e.g. DMG401026390), conformation stability by chaperones (e.g. DMG400008355) and degradation by proteases (e.g. DMG400028471) or the 26S proteasome pathway (see above). A number of genes encoding ‘chromo’ (chromatin organization modifier) domain containing proteins (e.g. DMG400043455) suggest that transcriptional regulation through chromatin structure and modification [55] contributes to the natural variation of TSC and TY. Chromatin organization might also be a biological explanation for the fact that several genes typical for retroelements such as ‘integrase core domain containing protein’ (e.g. DMG400015917) and ‘Gag-pol polyprotein’(e.g. DMG400038533) were found even among the 450 top candidate genes [56]. Alternatively, the effects observed at these loci might be indirectly caused by LD, as they were mostly located in central, heterochromatic chromosomal regions where LD is very extensive [53]. Numerous genes encoding structural components of the cell wall and the cytoskeleton as well as cell wall modifying enzymes also contained highly differential SNPs. Most remarkable in this category were two clusters of extensin genes on chromosome XII between 51 and 53 Mbp and between 60.7 and 60.8 Mbp, which are excellent candidates for the TSC-, TY- and TSY-QTL on the long arm of chromosome XII. Extensins have essential roles in building and maintaining the growing primary cell wall [57]. Transport processes seem to be important as well (e.g. DMG400022183). The category ‘biotic stress’ was represented by multiple genes with differential SNPs, such as putative genes for pathogen resistance (e.g. DMG400006800), the *PR1* gene (DMG400037874) and a cluster of methylketone synthase genes possibly involved in insect resistance [58] between 85.0 and 85.3 Mbp on chromosome I in QTL region 5. In the case of biotic stress it is clear that the effects on TY, TSC and TSY are indirect, caused by the obvious fact that susceptibility to pests and pathogens reduces crop yield.

## Conclusions

This is the first study, which physically dissects and maps QTL for potato tuber starch content, yield and starch yield based on genome wide, high troughput genotyping methods and the potato genome sequence. Comparative genotyping of three case-control populations by RAD sequencing and the 8.3 k SolCAP SNP array showed that 8303 SolCAP SNPs were not sufficient for the comprehensive tagging of QTL for TSC, TY and TSY in the potato genome, whereas 600,000 RADseq SNPs tagged most QTL redundantly due to LD within genes and between physically linked genes. Neither method captured fully the DNA diversity present in tetraploid potato cultivars. Comparative RAD sequencing of the QUEST case-control populations identified, depending on the criteria for data filtering, between 450 and 6664 annotated genes with differential SNPs for TSC, TY, TSY and combinations thereof. Based on a small sample of eleven genes, approximately half of the differential SNPs in 80% of the genes could be confirmed by association analysis in the unselected QUEST population. The 450 genes which contained highly differential SNPs causing amino acid changes, are considered as primary functional candidates for controlling the natural variation of tuber yield and starch content. TSC and TY are partially controlled by identical genes with pleotropic effects. In the majority of cases, a positive effect on yield was confounded by a negative effect on starch content and vice versa. However, 89 SNP alleles had synergistic effects on TY, TSY and TSY. These SNPs and the corresponding 72 genes are primary targets for the development of diagnostic markers for breeding applications. The positions of the genes with differential SNPs on the physical chromosome maps of potato suggest that at least 200 QTL regions with one or more underlying genes on all chromosomes control the natural variation of tuber starch content and yield, and consequently starch yield, in the QUEST population. GWAS for tuber starch content in the independent, unselected PIN184 population using the SolCAP SNP chip identified SNPs associated with TSC in 117 genes. The comparison of the physical QTL maps of the QUEST and the PIN184 population identified genomic regions, which harbour QTL for TSC, TY and TSY across genetic backgrounds and environments.

## Additional files

Additional file 1: Phenotypic values (adjusted means) of 90 cultivars of the QUEST population used for the assembly of case-control groups with high (1) and low (0) tuber starch content (TSC), yield (TY) and starch yield. (TSY) (XLSX 15 kb)

Additional file 2: Sequences, primers, annealing temperatures, SNP positions and nucleotide alleles of eleven genes fragments analysed in the QUEST population (*n* = 264) for association with TSC, TY and TSY. (DOCX 23 kb)

Additional file 3: Alleles and allele counts of differential RADseq SNPs (FDR < 0.05) in 6664 annotated genes in the QUEST case-control populations. Trait TSC is highlighted blue, TY orange and TSY green. SNPs differential for two traits are highlighted yellow. SNPs differential for all three traits are highlighted red. (XLSX 8035 kb)

Additional file 4: Alleles and allele counts of differential RADseq SNPs in 3144 annotated genes with at least one SNP at FDR < 0.001 in the QUEST case-control populations. Trait TSC is highlighted blue, TY orange and TSY green. SNPs differential for two traits are highlighted yellow. SNPs differential for all three traits are highlighted red. Loci highlighted dark green contain SNPs with FDR < 0.0001 and causing amino acid changes. (XLSX 2822 kb)

Additional file 5: Distribution of 3144 annotated genes with differential RADseq SNPs on the physical chromosome maps at a resolution of 0.5 Mbp. Intervals with at least 10 genes per 0.5 Mbp are highlighted yellow. (XLSX 37 kb)

Additional file 6: Alleles and allele counts of RADseq SNPs with differential allele frequencies (FDR < 0.05) in two case-control populations: TSC and TY, TSC and TSY or TY and TSY. Trait TSC is highlighted blue, TY orange and TSY green. (XLSX 1349 kb)

Additional file 7: Alleles and allele counts of RADseq SNPs with differential allele frequencies (FDR < 0.05) in all three case-control populations: TSC, TY and TSY. Trait TSC is highlighted blue, TY orange and TSY green. SNPs with unidirectional or neutral effect on the three traits are highlighted dark green. (XLSX 610 kb)

Additional file 8: Differential SolCAP SNPs in 275 genes in the QUEST case-control populations. Trait TSC is highlighted blue, TY orange and TSY green. (XLSX 84 kb)

Additional file 9: SolCAP SNPs in 117 genes associated with TSC in the PIN184 population. (XLSX 38 kb)

Additional file 10: Identical genes detected by differential SolCAP and RADseq SNPs in the QUEST case-control populations. (DOCX 24 kb)

Additional file 11: Identity of genes detected in previous association studies using the candidate gene approach with genes detected in this study. (DOCX 18 kb)

## Abbreviations

AFLP: Amplified fragment length polymorphism
BSA: Bulked segregant analysis
FDR: False discovery rate
GWAS: Genome wide association study
NBS-LRR: Nucleotide binding site-leucine rich repeat
PCR: Polymerase chain reaction
QTL: Quantitative trait locus
RADseq: Restriction-site associated DNA sequencing
RFLP: Restriction fragment length polymorphism
SNP: Single nucleotide polymorphism
TSC: Tuber starch content
TSY: Tuber starch yield
TY: Tuber yield.

## References

[1] Zeeman SC, Kossmann J, Smith AM (2010). Starch: its metabolism, evolution, and biotechnological modification in plants. Annu Rev Plant Biol.

[2] Storey M: The Harvested Crop. In: *Potato Biology and Biotechnology.* Edited by Vreugdenhil D, Bradshaw JE, Gebhardt C, Govers F, MacKerron DKL, Taylor MA, Ross HA. Amsterdam: Elsevier Science B.V.; 2007: 441–470.

[3] Isherwood FA (1973). Starch-sugar interconversion in *Solanum tuberosum*. Phytochemistry.

[4] Ellis RP, Cochrane MP, Dale MFB, Duffus CM, Lynn A, Morrison IM, Prentice RDM, Swanston JS, Tiller SA (1998). Starch production and industrial use. J Sci Food Agric.

[5] Simmonds NW (1977). Relations between specific gravity, dry matter content and starch content of potatoes. Potato Res.

[6] Bonierbale MW, Plaisted RL, Tanksley SD (1993). A test of the maximum heterozygosity hypothesis using molecular markers in tetraploid potatoes. Theor Appl Genet.

[7] Freyre R, Douches DS (1994). Development of a model for marker-assisted selection of specific gravity in diploid potato across environments. Crop Sci.

[8] Bradshaw JE, Hackett CA, Pande B, Waugh R, Bryan GJ (2008). QTL mapping of yield, agronomic and quality traits in tetraploid potato (*Solanum tuberosum* subsp. *tuberosum*). Theor Appl Genet.

[9] Manrique-Carpintero NC, Coombs JJ, Cui Y, Veilleux RE, Buell CR, Douches D (2015). Genetic map and QTL analysis of agronomic traits in a diploid potato population using single nucleotide polymorphism markers. Crop Sci.

[10] McCord PH, Sosinski BR, Haynes KG, Clough ME, Yencho GC (2011). Linkage mapping and QTL analysis of agronomic traits in tetraploid potato (*Solanum tuberosum* subsp. *tuberosum*). Crop Sci.

[11] Schäfer-Pregl R, Ritter E, Concilio L, Hesselbach J, Lovatti L, Walkemeier B, Thelen H, Salamini F, Gebhardt C (1998). Analysis of quantitative trait loci (QTLs) and quantitative trait alleles (QTAs) for potato tuber yield and starch content. Theor Appl Genet.

[12] Schönhals EM, Ortega F, Barandalla L, Aragones A. Ruiz de Galarreta JI, Liao J-C, Sanetomo R, Walkemeier B, Tacke E, Ritter E *et al*: identification and reproducibility of diagnostic DNA markers for tuber starch and yield optimization in a novel association mapping population of potato (*Solanum tuberosum* **L.)**. Theor Appl Genet. 2016;129(4):767–85. 10.1007/s00122-016-2665-7 PMC4799268 26825382

[13] Flint-Garcia SA, Thornsberry JM, Buckler ES (2003). Structure of linkage disequilibrium in plants. Annu Rev Plant Biol.

[14] Li L, Paulo MJ, Strahwald J, Lübeck J, Hofferbert HR, Tacke E, Junghans H, Wunder J, Draffehn A, van Eeuwijk F (2008). Natural DNA variation at candidate loci is associated with potato chip color, tuber starch content, yield and starch yield. Theor Appl Genet.

[15] Li L, Strahwald J, Hofferbert HR, Lubeck J, Tacke E, Junghans H, Wunder J, Gebhardt C (2005). DNA variation at the invertase locus *invGE/GF* is associated with tuber quality traits in populations of potato breeding clones. Genetics.

[16] Li L, Tacke E, Hofferbert H-R, Lübeck J, Strahwald J, Draffehn A, Walkemeier B, Gebhardt C (2013). Validation of candidate gene markers for marker-assisted selection of potato cultivars with improved tuber quality. Theor Appl Genet.

[17] Schreiber L, Nader-Nieto AC, Schönhals EM, Walkemeier B, Gebhardt C: SNPs in genes functional in starch-sugar interconversion associate with natural variation of tuber starch and sugar content of potato (*Solanum tuberosum* L.). *G3: Genes|Genomes|Genetics* 2014, 4(10):1797–1811. 10.1534/g3.114.012377 PMC4199688 25081979

[18] Urbany C, Stich B, Schmidt L, Simon L, Berding H, Junghans H, Niehoff K-H, Braun A, Tacke E, Hofferbert H-R (2011). Association genetics in *Solanum tuberosum* provides new insights into potato tuber bruising and enzymatic tissue discoloration. BMC Genomics.

[19] D’hoop BB, Keizer PLC, Paulo MJ, Visser RGF, van Eeuwijk FA, van Eck HJ (2014). Identification of agronomically important QTL in tetraploid potato cultivars using a marker–trait association analysis. Theor Appl Genet.

[20] Draffehn A, Meller S, Li L, Gebhardt C (2010). Natural diversity of potato (*Solanum tuberosum*) invertases. BMC Plant Biol.

[21] Fischer M, Schreiber L, Colby T, Kuckenberg M, Tacke E, Hofferbert H-R, Schmidt J, Gebhardt C (2013). Novel candidate genes influencing natural variation in potato tuber cold sweetening identified by comparative proteomics and association mapping. BMC Plant Biol.

[22] Li L, Paulo M-J, van Eeuwijk F, Gebhardt C (2010). Statistical epistasis between candidate gene alleles for complex tuber traits in an association mapping population of tetraploid potato. Theor Appl Genet.

[23] Frommer WB, Sonnewald U (1995). Molecular analysis of carbon partitioning in solanaceous species. J Exp Bot.

[24] Tetlow IJ, Morell MK, Emes MJ (2004). Recent developments in understanding the regulation of starch metabolism in higher plants. J Exp Bot.

[25] Regierer B, Fernie AR, Springer F, Perez-Melis A, Leisse A, Koehl K, Willmitzer L, Geigenberger P, Kossmann J (2002). Starch content and yield increase as a result of altering adenylate pools in transgenic plants. Nat Biotech.

[26] Zhang L, Häusler RE, Greiten C, Hajirezaei M-R, Haferkamp I, Neuhaus HE, Flügge U-I, Ludewig F (2008). Overriding the co-limiting import of carbon and energy into tuber amyloplasts increases the starch content and yield of transgenic potato plants. Plant Biotech J.

[27] Jonik C, Sonnewald U, Hajirezaei M-R, Flügge U-I, Ludewig F (2012). Simultaneous boosting of source and sink capacities doubles tuber starch yield of potato plants. Plant Biotech J.

[28] Navarro C, Abelenda JA, Cruz-Oro E, Cuellar CA, Tamaki S, Silva J, Shimamoto K, Prat S (2011). Control of flowering and storage organ formation in potato by FLOWERING LOCUS T. Nature.

[29] Kloosterman B, Abelenda JA. Gomez MdMC, Oortwijn M, de Boer JM, Kowitwanich K, Horvath BM, van Eck HJ, Smaczniak C, prat S *et al*: naturally occurring allele diversity allows potato cultivation in northern latitudes. Nature. 2013;495(7440):246–50. 10.1038/nature11912 23467094

[30] Chen H, Rosin FM, Prat S, Hannapel DJ (2003). Interacting transcription factors from the three-amino acid loop extension superclass regulate tuber formation. Plant Physiol.

[31] Sharma SK, Bolser D, de Boer J, Sønderkær M, Amoros W, Carboni MF, D’Ambrosio JM, de la Cruz G, Di Genova A, Douches DS *et al*: Construction of reference chromosome-scale pseudomolecules for potato: Integrating the potato genome with genetic and physical maps. *G3: Genes|Genomes|Genetics* 2013, **3**(11):2031–2047. 10.1534/g3.113.007153 PMC3815063 24062527

[32] PGSC (2011). Genome sequence and analysis of the tuber crop potato. Nature.

[33] Massa AN, Manrique-Carpintero NC, Coombs JJ, Zarka DG, Boone AE, Kirk WW, Hackett CA, Bryan GJ, Douches DS: Genetic Linkage Mapping of Economically Important Traits in Cultivated Tetraploid Potato (*Solanum tuberosum* **L.)**. *G3: Genes|Genomes|Genetics* 2015, **5**(11):2357–2364. 10.1534/g3.115.019646 PMC4632055 26374597

[34] Hamilton J, Hansey C, Whitty B, Stoffel K, Massa A, Van Deynze A, De Jong W, Douches D, Buell CR (2011). Single nucleotide polymorphism discovery in elite north american potato germplasm. BMC Genomics.

[35] Stich B, Urbany C, Hoffmann P, Gebhardt C (2013). Population structure and linkage disequilibrium in diploid and tetraploid potato revealed by genome-wide high-density genotyping using the SolCAP SNP array. Plant Breed.

[36] Mosquera T, Alvarez MF, Jiménez-Gómez JM, Muktar MS, Paulo MJ, Steinemann S, Li J, Draffehn A, Hofmann A, Lübeck J *et al*: Targeted and Untargeted Approaches Unravel Novel Candidate Genes and Diagnostic SNPs for Quantitative Resistance of the Potato (*Solanum tuberosum* L.) to *Phytophthora infestans* Causing the Late Blight Disease. *PLoS ONE* 2016, 11(6):e0156254. 10.1371/journal.pone.0156254 PMC4900573 27281327

[37] Hirsch CN, Hirsch CD, Felcher K, Coombs J, Zarka D, Van Deynze A, De Jong W, Veilleux RE, Jansky S, Bethke P *et al*: Retrospective View of North American Potato (*Solanum tuberosum* L.) Breeding in the 20th and 21st Centuries. *G3: Genes|Genomes|Genetics* 2013, **3**(6):1003–1013. 10.1534/g3.113.005595 PMC3689798 23589519

[38] Felcher KJ, Coombs JJ, Massa AN, Hansey CN, Hamilton JP, Veilleux RE, Buell CR, Douches DS (2012). Integration of two diploid potato linkage maps with the potato genome sequence. PLoS One.

[39] Davey JW, Blaxter ML (2010). RADSeq: next-generation population genetics. Briefings in Functional Genomics.

[40] Elshire RJ, Glaubitz JC, Sun Q, Poland JA, Kawamoto K, Buckler ES, Mitchell SE (2011). A robust, simple genotyping-by-sequencing (GBS) approach for high diversity species. PLoS One.

[41] Pajerowska-Mukhtar K, Stich B, Achenbach U, Ballvora A, Lübeck J, Strahwald J, Tacke E, Hofferbert H-R, Ilarionova E, Bellin D (2009). Single nucleotide polymorphisms in the *Allene Oxide Synthase 2* gene are associated with field resistance to late blight in populations of tetraploid potato cultivars. Genetics.

[42] Von Scheele C, Svensson G, Rasmusson J (1937). Die Bestimmung des Stärkegehalts und der Trockensubstanz der Kartoffel mit Hilfe des spezifischen Gewichts. Landw Vers Station.

[43] Etter P, Bassham S, Hohenlohe P, Johnson E, Cresko W: SNP discovery and genotyping for evolutionary genetics using RAD sequencing. In: *Molecular Methods for Evolutionary Genetics.* Edited by Orgogozo V, Rockman MV, vol. 772: Humana Press; 2011: 157–178. 10.1007/978-1-61779-228-1_9 PMC3658458 22065437

[44] Bus A, Hecht J, Huettel B, Reinhardt R, Stich B (2012). High-throughput polymorphism detection and genotyping in Brassica Napus using next-generation RAD sequencing. BMC Genomics.

[45] Langmead B, Trapnell C, Pop M, Salzberg SL: Ultrafast and memory-efficient alignment of short DNA sequences to the human genome. Genome Biol 2009, 10(3):R25-R25. 10.1186/gb-2009-10-3-r25 PMC2690996 19261174

[46] McKenna A, Hanna M, Banks E, Sivachenko A, Cibulskis K, Kernytsky A, Garimella K, Altshuler D, Gabriel S, Daly M (2010). The genome analysis toolkit: a MapReduce framework for analyzing next-generation DNA sequencing data. Genome Res.

[47] Cingolani P, Platts A, Wang LL, Coon M, Nguyen T, Wang L, Land SJ, Lu X, Ruden DM (2012). A program for annotating and predicting the effects of single nucleotide polymorphisms. SnpEff Fly.

[48] Voorrips R, Gort G, Vosman B (2011). Genotype calling in tetraploid species from bi-allelic marker data using mixture models. BMC Bioinformatics.

[49] Muktar MS, Lübeck J, Strahwald J, Gebhardt C. Selection and validation of potato candidate genes for maturity corrected resistance to *Phytophthora infestans* based on differential expression combined with SNP association and linkage mapping. Front Genet. 2015;6 10.3389/fgene.2015.00294 PMC4585299 26442110

[50] Mangeon A, Junqueira RM, Sachetto-Martins G (2010). Functional diversity of the plant glycine-rich proteins superfamily. Plant Signal Behav.

[51] Michelmore RW, Paran I, Kesseli RV (1991). Identification of markers linked to disease-resistance genes by bulked segregant analysis: a rapid method to detect markers in specific genomic regions by using segregating populations. Proc Natl Acad Sci U S A.

[52] Rickert AM, Kim JH, Meyer S, Nagel A, Ballvora A, Oefner PJ, Gebhardt C (2003). First-generation SNP/InDel markers tagging loci for pathogen resistance in the potato genome. Plant Biotechnol J.

[53] Vos PG, Paulo MJ, Voorrips RE, Visser RGF, van Eck HJ, van Eeuwijk FA. Evaluation of LD decay and various LD-decay estimators in simulated and SNP-array data of tetraploid potato. Theor Appl Genet. 2016:1–13. 10.1007/s00122-016-2798-8 PMC5214954 27699464

[54] Kofler R, Langmüller AM, Nouhaud P, Otte KA, Schlötterer C: Suitability of different mapping algorithms for genome-wide polymorphism scans with pool-seq data. *G3: Genes|Genomes|Genetics* 2016. 10.1534/g3.116.034488 PMC5100849 27613752

[55] Eissenberg JC (2012). Structural biology of the chromodomain: form and function. Gene.

[56] von Sternberg R, Shapiro JA (2005). How repeated retroelements format genome function. Cytogenet Genome Res.

[57] Lamport DTA, Kieliszewski MJ, Chen Y, Cannon MC (2011). Role of the extensin superfamily in primary cell wall architecture. Plant Physiol.

[58] Yu G, Nguyen TTH, Guo Y, Schauvinhold I, Auldridge ME, Bhuiyan N, Ben-Israel I, Iijima Y, Fridman E, Noel JP (2010). Enzymatic functions of wild tomato methylketone synthases 1 and 2. Plant Physiol.

[59] Milligan G, Kostenis E (2006). Heterotrimeric G-proteins: a short history. Br J Pharmacol.

[60] Laity JH, Lee BM, Wright PE (2001). Zinc finger proteins: new insights into structural and functional diversity. Curr Opin Struct Biol.

[61] Cha J-Y, Barman DN, Kim MG, Kim W-Y (2015). Stress defense mechanisms of NADPH-dependent thioredoxin reductases (NTRs) in plants. Plant Signal Behav.

[62] Shankar A, Agrawal N, Sharma M, Pandey A, Pandey GK (2015). Role of protein tyrosine phosphatases in plants. Curr Genomics.

[63] Tang C, Wang X, Duan X, Wang X, Huang L, Kang Z (2013). Functions of the lethal leaf-spot 1 gene in wheat cell death and disease tolerance to Puccinia striiformis. J Exp Bot.

[64] Wilkens S: Structure and mechanism of ABC transporters. *F1000Prime Rep 2015, 7:14.* 10.12703/P7-14 PMC4338842 25750732

[65] Zarepour M, Simon K, Wilch M, Nieländer U, Koshiba T, Seo M, Lindel T, Bittner F (2012). Identification of superoxide production by *Arabidopsis thaliana* aldehyde oxidases AAO1 and AAO3. Plant Mol Biol.

[66] Van der Hoorn RAL (2008). Plant proteases: from phenotypes to molecular mechanisms. Annu Rev Plant Biol.

[67] Lu W, Tang X, Huo Y, Xu R, Qi S, Huang J, Zheng C (2012). Wu C-a: **identification and characterization of fructose 1,6-bisphosphate aldolase genes in Arabidopsis reveal a gene family with diverse responses to abiotic stresses**. Gene.

[68] Howard TP, Fryer MJ, Singh P, Metodiev M, Lytovchenko A, Obata T, Fernie AR, Kruger NJ, Quick WP, Lloyd JC (2011). Antisense suppression of the small chloroplast protein CP12 in tobacco alters carbon partitioning and severely restricts growth. Plant Physiol.

[69] Bouton S, Leboeuf E, Mouille G, Leydecker M-T, Talbotec J, Granier F, Lahaye M, Höfte H, Truong H-N (2002). QUASIMODO1 encodes a putative membrane-bound glycosyltransferase required for normal pectin synthesis and cell adhesion in Arabidopsis. Plant Cell.

[70] Barkan A, Small I (2014). Pentatricopeptide repeat proteins in plants. Annu Rev Plant Biol.

[71] Qi W, Yang Y, Feng X, Zhang M, Song R. Mitochondrial function and maize kernel development requires Dek2, a pentatricopeptide repeat protein involved in nad1 mRNA splicing. Genetics. 2016; 10.1534/genetics.116.196105 PMC5223505 27815362

[72] Tchórzewski M (2002). The acidic ribosomal P proteins. Int J Biochem Cell Biol.

[73] Hafrén A, Eskelin K, Mäkinen K (2013). Ribosomal protein P0 promotes potato virus a infection and functions in viral translation together with VPg and eIF(iso)4E. J Virol.

[74] Hansen SF, Harholt J, Oikawa A, Scheller HV (2012). Plant glycosyltransferases beyond CAZy: a perspective on DUF families. Front Plant Sci.

[75] Le Roy J, Huss B, Creach A, Hawkins S, Neutelings G (2016). Glycosylation is a major regulator of phenylpropanoid availability and biological activity in plants. Front Plant Sci.

[76] Rasbery JM, Hui Shan H, LeClair JM, Norman M, Matsuda SPT, Bartel B (2007). *Arabidopsis thaliana* squalene epoxidase 1 is essential for root and seed development. J Biol Chem.

[77] Barbier-Brygoo H, Vinauger M, Colcombet J, Ephritikhine G, Frachisse J-M, Maurel C (2000). Anion channels in higher plants: functional characterization, molecular structure and physiological role. Biochim Biophys Acta Biomembr.

[78] Palmgren MG (2001). PLANT PLASMA MEMBRANE H+−ATPases: powerhouses for nutrient uptake. Annu Rev Plant Physiol Plant Mol Biol.

[79] Hardie DG (1999). Plant protein serine/threonine kinases: classification and functions. Ann Rev Plant Physiol Plant Mol Biol.

[80] Gabriëls SHEJ, Vossen JH, Ekengren SK. Ooijen Gv, Abd-el-Haliem AM, berg GCMvd, Rainey DY, Martin GB, Takken FLW, wit PJGMd *et al*: an NB-LRR protein required for HR signalling mediated by both extra- and intracellular resistance proteins. Plant J. 2007;50(1):14–28. 10.1111/j.1365-313X.2007.03027.x 17346268

[81] Ray A, Robinson-Beers K, Ray S, Baker SC, Lang JD, Preuss D, Milligan SB, Gasser CS (1994). Arabidopsis floral homeotic gene BELL (BEL1) controls ovule development through negative regulation of AGAMOUS gene (AG). Proc Natl Acad Sci U S A.

[82] Guo J, Zhou W, Lu Z, Li H, Li H, Gao F (2015). Isolation and functional analysis of chalcone isomerase gene from purple-fleshed sweet potato. Plant Mol Biol Rep.

[83] Athauda Senarath BP, Matsumoto K, Rajapakshe S, Kuribayashi M, Kojima M, Kubomura-Yoshida N, Iwamatsu A, Shibata C, Inoue H, Takahashi K (2004). Enzymic and structural characterization of nepenthesin, a unique member of a novel subfamily of aspartic proteinases. Biochem J.

[84] Noguero M, Atif RM, Ochatt S, Thompson RD (2013). The role of the DNA-binding one zinc finger (DOF) transcription factor family in plants. Plant Sci.

[85] Wang L, Ouyang M, Li Q, Zou M, Guo J, Ma J, Lu C, Zhang L (2010). The Arabidopsis chloroplast ribosome recycling factor is essential for embryogenesis and chloroplast biogenesis. Plant Mol Biol.

[86] Martín I, Dopico B, Muñoz FJ, Esteban R, Oomen RJFJ, Driouich A, Vincken J-P, Visser R, Labrador E (2005). In vivo expression of a Cicer Arietinum β-galactosidase in potato tubers leads to a reduction of the galactan side-chains in cell wall pectin. Plant Cell Physiol.

