# Supplementary material for: Physical mapping of QTL for tuber yield, starch content and starch yield in tetraploid potato (Solanum tuberosum L.) by means of genome wide genotyping by sequencing and the 8.3 K SolCAP SNP array
Source: BMC Genomics. 2017 Aug 22;18:642. doi: 10.1186/s12864-017-3979-9 (PMC5567664; doi:10.1186/s12864-017-3979-9)
Supplement: Supplementary file 2 — Sequences, primers, annealing temperatures, SNP positions and nucleotide alleles of eleven genes fragments analysed in the QUEST population (n = 264) for association with TSC, TY and TSY. (DOCX 23 kb) [file 12864_2017_3979_MOESM2_ESM.docx]

**Additional file 10.** Identical genes detected by differential SolCAP and RADseq SNPs in the QUEST case-control populations

| Locus PGSC0003 | Chromosome:  Mbp (v4.03) | Annotation | SolCAP SNPs | RADseq SNPs | Function category |
| --- | --- | --- | --- | --- | --- |
| DMG400006816 | I:64.2 | ATP binding protein, kinase | TSC | TSC, TY | Signalling, regulation (IPR017441) ^1^ |
| DMG400006800 | I:65.8 | NBS-LRR protein | TSC | TSC, TY, TSY | Biotic stress |
| DMG401025958 | I:70.6 | Glycine-rich protein | TSY, TY, TSC | TSC, TY | Multiple functions |
| DMG400027768 | I:71.1 | COP11 protein | TSY | TSY, TY | Photomorphogenesis |
| DMG401000076 | I:72.8 | ABIL1 | TY, TSY | TSC, TY, TSY | Cytoskeleton (uniprot/Q8S8M5) ^2^ |
| DMG400022473 | I:75.5 | Zeta-carotene desaturase | TSC | TSC, TY, TSY | Carotenoid biosynthesis |
| DMG400028611 | I:76.5 | Peroxisomal membrane protein 34 | TSC | TSC | Transport (uniprot/O43808) |
| DMG400024755 | I:77.3 | Xyloglucan endotransglucosylase/hydrolase1 | TSY | TSC, TY | Cell wall biosynthesis (uniprot/Q40144) |
| DMG400003102 | I:83.0 | L-galactose dehydrogenase | TY, TSY | TSC, TY, TSY | Ascorbate biosynthesis |
| DMG400000724 | I:85.9 | Serine/threonine protein kinase family | TY, TSY | TSC, TY | Posttranslational regulation |
| DMG400030951 | I:86.3 | Ubiquitin-activating enzyme E1b | TSC | TSC, TSY | Protein degradation by 26S proteasome |
| DMG400009417 | II:27.1 | Kinase | TSC | TSC, TSY | Phosphorylation of specific substrates (proteins, lipids, carbohydrates and others) |
| DMG400009705 | II:30.3 | Purine transporter | TY, TSY, TSC | TY, TSY | Transport |
| DMG400010718 | II:32.8 | GDSL-motif lipase/hydrolase family protein | TY, TSC | TY | Lipid metabolism |
| DMG401017733 | II:37.4 | Zinc finger protein | TSC | TSC, TY, TSY | Multiple regulatory functions |
| DMG400026392 | II:41.4 | Mannose-6-phosphate isomerase | TSC, TY | TSC, TY | Carbohydrate metabolism (uniprot/P34949) |
| DMG400043061 | II:42.5 | Serine-Threonine protein kinase, plant-type | TY | TY, TSC | Posttranslational regulation |
| DMG400004020 | II:43.8 | Zeaxanthin epoxidase, chloroplastic | TY | TY, TSY | Abscisic acid biosynthesis (uniprot/P93236) |
| DMG400024561 | III:55.6 | Serin-Threonine protein kinase | TSC | TSC | Posttranslational regulation |
| DMG400000619 | III:56.6 | Nucleosome-binding protein | TSC | TSC, TY | Chromatin structure |
| DMG400000639 | III:56.6 | Delta-9 desaturase | TSC | TSC, TY | Lipid metabolism |
| DMG400025300 | IV:3.9 | Conserved gene of unknown function | TSC, TSY | TSC, TY | Unknown |
| DMG400009981 | IV:71.6 | 1,4-alpha-glucan branching enzyme | TSC | TSC, TY, TSY | Starch biosynthesis (uniprot/P30924) |
| DMG400010007 | IV:72.0 | Pyrophosphate-fructose 6-phosphate 1-phosphotransferase subunit alpha | TSC | TSC, TY, TSY | Glycolysis, gluconeogenesis (uniprot/P21342) |
| DMG400014542 | V:2.8 | Conserved gene of unknown function | TY | TSC, TY, TSY | Unknown |
| DMG400031262 | V:5.0 | Methyltransferase | TSC, TSY | TSC, TY, TSY | Methylation of specific substrates |
| DMG400030978 | V:8.2 | Polygalacturonase non-catalytic subunit AroGP2 | TSC | TSC, TY | Cell wall, carbohydrate metabolism (uniprot/P93217) |
| DMG400021635 | V:43.7 | Conserved gene of unknown function | TY, TSY | TSC, TSY | Unknown |
| DMG400008445 | V:49.7 | 60S ribosomal protein L6 | TSY | TSC, TY, TSY | Protein biosynthesis |
| DMG400008462 | V:49.7 | E3 ubiquitin-protein ligase UPL5 | TSC | TY | Protein degradation by 26S proteasome (uniprot/Q9SU29) |
| DMG401023454 | V:51.8 | Biotin carboxylase carrier protein | TSC, TY | TSC, TY | Lipid metabolism |
| DMG400007297 | VI:0.31 | Protein phosphatase-2C | TSC, TY, TSY | TSC, TY | Posttranslational regulation |
| DMG402007274 | VI:0.37 | Isoamylase isoform 3, debranching enzyme | TSC | TSC, TY, TSY | Starch metabolism |
| DMG402017934 | VI:0.41 | Pectinesterase | TSC | TSC, TY, TSY | Cell wall metabolism (IPR000070) |
| DMG400009689 | VI:6.4 | Protein kinase | TSC, TSY | TY, TSY | Posttranslational regulation |
| DMG400016296 | VI:39.5 | O-linked n-acetylglucosamine transferase (OGT) | TY, TSY | TSC, TY | Posttranslational regulation |
| DMG400027936 | VII:3.9 | Sucrose-phosphate-synthase | TY, TSY | TY, TSY | Carbohydrate metabolism |
| DMG400028958 | VII:46.8 | Multicopper oxidase | TSC | TSC | Oxidation |
| DMG400009380 | VII:49.7 | Leucine-rich repeat receptor kinase CLAVATA1 | TSY | TSC, TY, TSY | Regulation, development |
| DMG400017292 | VII:50.9 | F-box protein POF4, elongin-A | TSC | TSC | Transcriptional regulation (uniprot/O59671) |
| DMG400031084 | VII:51.4 | Glucose-1-phosphate adenylyltransferase small subunit, chloroplastic/amyloplastic | TSC | TSC | Starch synthesis |
| DMG400031099 | VII:51.5 | Endosomal P24A protein | TSC | TSC, TY, TSY | Vesicle transport |
| DMG400007070 | VII:52.5 | Polynucleotide kinase-3'-phosphatase | TSC | TSC, TY, TSY | DNA repair (uniprot/Q96T60) |
| DMG400007390 | VIII:40.7 | Beta-ketoacyl-CoA synthase | TSC | TSC, TSY | Lipid metabolism (IPR018201) |
| DMG400010842 | VIII:43.4 | Conserved gene of unknown function | TY, TSY | TSC, TSY | Unknown |
| DMG400008891 | IX:1.4 | GDP-D-mannose pyrophosphorylase | TSC, TY | TSC, TY | Ascorbic acid biosynthesis, cell wall |
| DMG400029885 | IX:19.2 | Kinase | TSC | TSC, TY | Phosphorylation of specific substrates (proteins, lipids, carbohydrates and others) |
| DMG400027986 | IX: 33.7 | Copper ion binding protein | TSC | TSC, TY | Copper ion transport (IPR000428) |
| DMG400011395 | IX: 52.4 | Chloroplast protein import component Toc159 | TSC, TSY | TY | Chloroplast protein transport |
| DMG400014421 | X:1.2 | Proline synthetase associated protein | TY, TSY | TSC, TSY | Prolin metabolism, abiotic stress |
| DMG400025001 | X:1.7 | Global transcription factor group | TY, TSY | TSC, TY, TSY | Transcription regulation |
| DMG400019409 | X:6.3 | Chromatin remodeling complex subunit | TSC, TSY | TSC, TY, TSY | Chromatin structure |
| DMG400011070 | X:54.3 | FHY1 | TSC | TSC, TY, TSY | Signalling |
| DMG400028261 | X:55.6 | Fructose-bisphosphate aldolase | TSC | TSC, TSY | Glycolysis, gluconeogenesis, Calvin cycle |
| DMG400023687 | X:55.7 | Ribosomal protein S9 | TSY | TY | Protein synthesis |
| DMG400013259 | XI:0.9 | Nonsense-mediated mRNA decay protein | TY, TSY | TSC, TY, TSY | mRNA degradation |
| DMG400015693 | XI:1.6 | Resistance gene | TSC | TSC | Biotic stress |
| DMG400016208 | XI:2.5 | Phosphofructokinase family protein | TSC, TY | TSC, TY | Glycolysis, carbohydrate metabolism (IPR000023) |
| DMG400016219 | XI:2.6 | GTP cyclohydrolase II | TSC, TY | TSC, TY, TSY | Riboflavin biosynthesis (IPR000926) |
| DMG400031071 | XI:4.0 | No apical meristem (Nam) 9 | TSC | TSC, TY, TSY | Development |
| DMG400000988 | XI:6.4 | Synovial sarcoma associated ss18 protein | TY, TSY | TSC, TY, TSY | Transcriptional regulation |
| DMG400003985 | XI:7.7 | Actin | TY, TSY | TSC | Cytoskeleton |
| DMG400009240 | XI:8.8 | CSN5 protein | TSC | TSC, TY | Signalling |
| DMG400019677 | XI:42.0 | Elongation factor 1-alpha | TY | TY | Protein synthesis |
| DMG400015379 | XII:0.3 | Malate dehydrogenase | TSC | TSC, TY, TSY | Tricarboxylic acid cycle (IPR001507) |
| DMG400015368 | XII:0.5 | Conserved gene of unknown function | TSC | TSC, TY | Unknown |
| DMG400007797 | XII:1.3 | Citrate synthase | TY | TSC, TY | Tricarboxylic acid cycle (uniprot/Q43175) |
| DMG400007821 | XII:2.0 | Endosomal P24A protein | TSC | TSC, TY, TSY | Vesicle transport |
| DMG400029787 | XII:10.2 | Molecular chaperone Hsp90-1 | TY | TSC, TY | Protein folding, stress response |
| DMG400028851 | XII:53.0 | Translation initiation factor eif-2b alpha subunit | TSY | TSC, TY, TSY | Protein synthesis, virus infection? |
| DMG400029407 | XII:57.5 | Conserved gene of unknown function | TY, TSY | TY | Unknown |
| DMG400004280 | XII:61.1 | Phytocalpain | TSC | TSC, TY, TSY | Development |

^1^ Entry in the InterPro database (<http://www.ebi.ac.uk/interpro/>)

^2^ Entry in the uniprot database (http://www.uniprot.org/)
