# Supplementary material for: Physical mapping of QTL for tuber yield, starch content and starch yield in tetraploid potato (Solanum tuberosum L.) by means of genome wide genotyping by sequencing and the 8.3 K SolCAP SNP array
Source: BMC Genomics. 2017 Aug 22;18:642. doi: 10.1186/s12864-017-3979-9 (PMC5567664; doi:10.1186/s12864-017-3979-9)
Supplement: Supplementary file 3 — Alleles and allele counts of differential RADseq SNPs (FDR < 0.05) in 6664 annotated genes in the QUEST case-control populations. Trait TSC is highlighted blue, TY orange and TSY green. SNPs differential for two traits are highlighted yellow. SNPs differential for all three traits are highlighted red. (XLSX 8035 kb) [file 12864_2017_3979_MOESM3_ESM.docx]

**Additional file 11.** Identity of genes detected in previous association studies using the candidate gene approach with genes detected in this study**.** Thirty nine candidate genes associated with TSC, TY and/or TSY in previous association studies were interrogated, whether or not they were detected in the present study by genome wide SNP genotyping in the QUEST case-control and PIN184 populations.

| Locus PGSC0003 | Gene (acronym or marker name) | Chromosome: Mbp (v4.03) | Detected in QUEST cases/controls and PIN184 population: traits | Reference ^2^ |
| --- | --- | --- | --- | --- |
| DMG400000735 | ADP-glucose pyrophosphorylase (*AGPaseS*) | I:86.1 | no | 3, 6, 8, 9 |
| DMG400008322 | Starch synthase IV (*SS IV*) | II:30.1 | no | 9 |
| DMG400031758 | Lipase class III (*LIP III*) | II:33.0 | In QUEST by RADseq (1 SNP * ^1^): TY, TSY | 5 |
| DMG400004020 | Zeaxanthin epoxidase (*ZEP*) | II:43.8 | In QUEST by RADseq (6 SNPs *): TY, TSY; and by solcap_snp_c2_27218: TY | 5 |
| DMG400010074 | Hydroxyproline-rich glycoprotein family protein (*StI024*) | II:44.5 | no | 5 |
| DMG400020269 | Glucose-6-phosphate dehydrogenase (*G6pdh*) | II:47.7 | no | 2 |
| Not on physical map | Starch phosphorylase 1a (*PHO1A, Stp23*) | III:? | In both QUEST and PIN184 by solcap_snp_c2_21313: TSC | 2, 5, 6, 8 |
| DMG400013856 | Soluble acid invertase (*Pain-1*) | III:39.2 | no | 2, 3, 4, 6, 9 |
| Not annotated | Phosphoglucomutase (*PGM*) | III:41.5 | Not tested in QUEST by RADseq | 8 |
| DMG400010119 | Fibrillarin homolog (*StI013*) | III:43.7 | In QUEST by RADseq (3 SNPs *): TSC, TY | 5 |
| DMG402018552 | Soluble starch synthase I (*SssI*) | III:45.9 | In QUEST by RADseq (3 SNPs): TSC, TSY | 2, 8 |
| DMG400003155 | 4-coumarate CoA ligase (*4Cl*) | III:47.1 | no | 5 |
| DMG400010146 | Kunitz-type invertase inhibitor (*KT_InvInh*) | III:49.4 | no | 7 |
| DMG400009178 | Pectinesterase (*PEST*) | III:61.8 | In QUEST by RADseq (1 SNP): TSC | 5 |
| DMG400012910 | Glucose-6-phosphate isomerase 1 (*PGI1*) | IV:64.8 | no | 9 |
| DMG400028382 | Starch phosphorylase 1b (*PHO1B, StpL*) | V:0.3 | no | 2, 5, 6, 8 |
| DMG400018408 | Cyclic DOF factor 1 (*StCDF1*) | V:4.5 | no | 9 |
| DMG400025610 | Sucrose transporter (*Sut2*) | V:5.7 | no | 2 |
| DMG400007677 | Glucan water dikinase (*GWD*) | V:9.8 | In QUEST by RADseq (7 SNPs *): TSC, TSY | 8 |
| DMG400033034 | Plasma membrane H^+^ ATPase 1 (*PHA1*) | VI:51.9 | In QUEST by RADseq (4 SNPs): TSC, TY, TSY; and in PIN184 by solcap_snp_c2_8867: TSC | 5 |
| DMG400011189 | Hydroxycinnamoyl quinate CoA transferase (*HQT*) | VII:1.0 | no | 5 |
| DMG400027936 | Sucrose phosphate synthase (*SPS*) | VII:3.9 | In QUEST by RADseq (2 SNPs): TSC, TY; and solcap_snp_c2_55832: TY, TSY | 2 |
| DMG400004101 | Plasma membrane H^+^ ATPase 2 (*PHA2*) | VII:10.8 | In QUEST by RADseq (5 SNPs *): TSC, TY | 2 |
| DMG400015952 | ADP-glucose pyrophosphorylase (*AGPaseB*) | VII:21.4 | no | 2, 5 |
| DMG400008205 | C2H2L domain class transcription factor (*SSR327*) | VIII:35.5 | no | 5 |
| Intergenic | GP171 | VIII:37.5 | Not tested in QUEST by RADseq | 2 |
| DMG400029575 | Polyphenol oxidase (*PPO*) | VIII:45.6 | In QUEST by RADseq (2 SNPs): TSC | 5 |
| DMG400001855 | Plastidic beta-amylase 1 (*BMY1*) | VIII:50.6 | In QUEST by RADseq (3 SNPs *): TSC, TY | 8 |
| DMG400004790 | Invertase (*Inv-8/2*) | VIII:52.7 | In QUEST by RADseq (7 SNPs *): TSC, TY, TSY | 8 |
| DMG400008942/43 | Apoplastic invertase (*Inv-GE/GF*) | IX:2.5 | no | 1, 2 |
| DMG400016613 | Phosphoglucan water dikinase (*PWD*) | IX:60.6 | In QUEST by RADseq (6 SNPs *): TSC, TY | 8, 9 |
| DMG400019149 | Ribulose bisphosphate carboxylase/oxygenase activase (*Rca*) | X:50.9 | In QUEST by RADseq (4 SNPs): TSC | 4, 9 |
| DMG401028252 | Apoplastic invertase (*InvCD111/141*) | X:55.8 | no | 2, 3, 9 |
| DMG400008132 | L-galactono-1,4-lactone dehydrogenase (*GLDH*) | X:58.3 | no | 5 |
| DMG400007178 | p-coumarate 3-hydroxylase (C3H) | X:59.5 | no | 5 |
| Not annotated | Debranching enzyme (DBE) | XI:3.9 | Not tested in QUEST by RADseq | 2 |
| Intergenic | Microsatellite STM0037 | XI:8.2 | Not tested in QUEST by RADseq | 2, 5, 9 |
| DMG400006458 | Leucine rich repeat protein (*SSR20*) | XII:11.3 | no | 5 |
| DMG400007831 | Leucine amino peptidase (*LAP*) | XII:2.3 | no | 7, 9 |

^1^ * indicates that at least one differential SNP caused an amino acid change

^2^ 1 = Li L, Strahwald J, Hofferbert HR, Lubeck J, Tacke E, Junghans H, Wunder J, Gebhardt C: **DNA variation at the invertase locus *invGE/GF* is associated with tuber quality traits in populations of potato breeding clones**. *Genetics* 2005, **170**(2):813-821.

2 = Li L, Paulo MJ, Strahwald J, Lübeck J, Hofferbert HR, Tacke E, Junghans H, Wunder J, Draffehn A, van Eeuwijk F *et al*: **Natural DNA variation at candidate loci is associated with potato chip color, tuber starch content, yield and starch yield**. *Theor Appl Genet* 2008, **116**:1167-1181.

3 = Draffehn A, Meller S, Li L, Gebhardt C: **Natural diversity of potato (*Solanum tuberosum*) invertases**. *BMC Plant Biol* 2010, **10**(1):271.

4 = Li L, Paulo M-J, van Eeuwijk F, Gebhardt C: **Statistical epistasis between candidate gene alleles for complex tuber traits in an association mapping population of tetraploid potato**. *Theor Appl Genet* 2010, **121**(7):1303-1310.

5 = Urbany C, Stich B, Schmidt L, Simon L, Berding H, Junghans H, Niehoff K-H, Braun A, Tacke E, Hofferbert H-R *et al*: **Association genetics in *Solanum tuberosum* provides new insights into potato tuber bruising and enzymatic tissue discoloration**. *BMC Genomics* 2011, **12**(1):7.

6 = Li L, Tacke E, Hofferbert H-R, Lübeck J, Strahwald J, Draffehn A, Walkemeier B, Gebhardt C: **Validation of candidate gene markers for marker-assisted selection of potato cultivars with improved tuber quality**. *Theor Appl Genet* 2013, **126**(4):1039-1052.

7 = Fischer M, Schreiber L, Colby T, Kuckenberg M, Tacke E, Hofferbert H-R, Schmidt J, Gebhardt C: **Novel candidate genes influencing natural variation in potato tuber cold sweetening identified by comparative proteomics and association mapping**. *BMC Plant Biol* 2013, **13**(1):113.

8 = Schreiber L, Nader-Nieto AC, Schönhals EM, Walkemeier B, Gebhardt C: **SNPs in genes functional in starch-sugar interconversion associate with natural variation of tuber starch and sugar content of potato (*Solanum tuberosum* L.)**. *G3: Genes|Genomes|Genetics* 2014, **4**(10):1797-1811.

9 = Schönhals EM, Ortega F, Barandalla L, Aragones A, Ruiz de Galarreta JI, Liao J-C, Sanetomo R, Walkemeier B, Tacke E, Ritter E *et al*: **Identification and reproducibility of diagnostic DNA markers for tuber starch and yield optimization in a novel association mapping population of potato (*Solanum tuberosum* L.)**. *Theor Appl Genet* 2016, **129**(4):767-785.
