# Supplementary material for: Physical mapping of QTL for tuber yield, starch content and starch yield in tetraploid potato (Solanum tuberosum L.) by means of genome wide genotyping by sequencing and the 8.3 K SolCAP SNP array
Source: BMC Genomics. 2017 Aug 22;18:642. doi: 10.1186/s12864-017-3979-9 (PMC5567664; doi:10.1186/s12864-017-3979-9)
Supplement: Supplementary file 4 — Alleles and allele counts of differential RADseq SNPs in 3144 annotated genes with at least one SNP at FDR < 0.001 in the QUEST case-control populations. Trait TSC is highlighted blue, TY orange and TSY green. SNPs differential for two traits are highlighted yellow. SNPs differential for all three traits are highlighted red. Loci highlighted dark green contain SNPs with FDR < 0.0001 and causing amino acid changes. (XLSX 2822 kb) [file 12864_2017_3979_MOESM4_ESM.docx]

**Additional file 2**: Genomic fragments of eleven genes analysed in the QUEST population (n = 264) for SNPs by amplicon sequencing or pyrosequencing. SNP positions, nucleotide alleles and identifications are included. Sequences were retrieved from pseudomolecules (v4.03) at <http://potato.plantbiology.msu.edu/cgi-bin/gbrowse/potato/>. PCR primers are underlined. SNP’s are identified by the last four digits of their position on the pseudomolecules (v4.03) or by SolCAP SNP identifiers. SNPs differential in the QUEST case-control populations are highlighted yellow.

***AOX,*** Aldehyde Oxidase (PGSC0003DMG401006826)

Chr01:64545289..64544510

GGGATTCTCTAAGCTTACTTCGTCCGAAGCTGAAAAGGCAATAGAAGGAAACCTTTGTCGGT

.5214 .5193

GCACTGGATACCG(G/A/T)CCCATTGCTGATGCCTGCAA(G/A)ACTTTTGCTGCTGATAT

.5166 .5161 .5136

TGATATAGA(G/A)GATT(G/T)GGGATTCAATGCTTTTTGGAAAAA(C/T/G)GGAGATTC

.5118 .5090 .5080

CAAGGAAAT(G/T)AAAGTAAGTAAATTACCTCCCTATGAT(A/C)CAACTAAAA(A/T)CT

.5049

TCAATACATATCCCGAGTTCTTGAAAAG(T/G)GAATCCACCACGAATTTAGACTCTTCAAG

.5014 .4985 .4979

GTACC(C/T)TTGGTATACTCCTGTTTCCATTGAGGAT(C/T)TACAG(G/A)GCTTGTTGA

.4929

ACTCCAACGTGACAGAAAATGGTGCAAGCTTTAAACTAGT(C/T)GTTGGTAATACTGGAAC

.4853

AGGTTATTATAAAGAAACTCAGCGATATGATCACTATATTGATCTCAGGTACGTTCCC(G/A

)AACTCTCGATCATCAAAAGAGATCAGGCAGGCATCGACATTGGATCAACTGTGACAATCTA

.4779 .4742(del)

TAAACTCATTTC(A/G)TTCTTGAAAGAGGAAAGTAAAATCAATTTCGGTTCA(TATGGGA)

.4680

AGTTGGTGTCCGATAAACTGGCTTACCACATGGAGAAGATTGCTTCACCATTTGT(T/G)AG

AAATTCTGCTAGTGTGGGAGGAAATTTGGTTATGGCACAGAAGAATGGTTTTCCTTCAGATA

TAGCTACATTATTTCTTGGGCTTTGTGCTACTGTTAGCTTGATGACTCGTCATGGACTTGTA

AAGCTCACATGGGAGGAGTTATTGTCGAAACCACCACTGGACTC

Amplicon sequencing: T_annealing_ = 62°C

***ADXR (Nadph)*** NADPH: adrenodoxin oxidoreductase, mitochondrial (PGSC0003DMG400018273)

Chr01:79205531..79205742

CCTTTTGAGAAAAGTGAAGCCTATCTTGTTGAAAAGCTGATTGGATGTGTTAGAGACTTGGG

GTCCTAGATGGTGTGAGGAGTTGTCTTCTTACTTCCTCCATTTTAATTTGTTCGTCTTTTTC

.5701 .5705

TCCTTTTAGTTTATTTAAAAAAGAAAGCCTCTTTCCCAATTTGACA(G/T)CTC(T/C)TTA

.5710

A(C/T)TCCCACATCCAACATGACATATTTAAGGCCAC

Pyrosequencing: T_annealing_ = 63°C

Biotinylated primer: CCTTTTGAGAAAAGTGAAGCCTATCT

Sequencing primer: CATGTTGGATGTGGGA

***CP5,*** Cysteine protease Cp5 (PGSC0003DMG400010234)

Chr02:32062634..32063098

GCCATTTTTCTCTTCCTTGCTTCCACTCCAATTTTGTCTCCATGATCCTGCAAAAAAAAGGA

AAGAAATAATCAAACAAATCATAATTTATTTAAATTTCTAAAGATTTGTTCTATCTGTATTA

TTTGGGACCTTTTTCTATCACATTGAATATTTAATCTTTGGTAGAAAGCGTTTTGCTCTGAT

ATATTTGGTGTAGGAAGCGATAAGCGATTAGTTGGACCGGTTGATTTCAGGATGTTTAAAGC

AAAGCGAAAATTATTTACTCTTTGATTTATCCAAAGTGAGTTATTTTAGGCATTGTACTATT

TTCTGATGGTATCTAATAATTGAGCAAAATTTTGCAACCTTAAATATCTTTCAAACCCTTTT

.3041

GCCTTGCTTTTAGTCTGTAAAGATTACTCCTAGTA(C/T)CTTGTTGATGATGACAAATCTA

ATCTATTTAGCATGGTACCTCCAACTGCCGCAAAT

Pyrosequencing: T_annealing_ = 55°C

Biotinylated primer: GCCATTTTTCTCTTCCTTGCTTC

Sequencing primer: ATTTGTCATCATCAACAAG

***SpRMD5,*** Sporulation protein RMD5 (PGSC0003DMG400030664)

Chr02:42087526..42087883

AAATCCCCAAGGGCCTGTGTGTGCACAGTTAGAATACAAGATGAGTAAAACAATACACCATG

ATCATAAATCTGCAAAAATTACATTAATTGGTAGAAGAGATAAGAAGTGACATCGCAAGACT

GATTCAGATGCTGCATCTAGACCACACTAATCTAATGCAGCAATCTCAAGATTGGGGGTACT

ATCCTTCTTGGTAAATATTTTTTTCCTAATGACTTGCACAAAAGTAATTTCATGGAAGGGAC

.7802 .7811

ACTATGGATGCAGATTTCAATGAAACTG(T/G)AATATGTA(T/C)GCCTAATGCTTCGGTA

CCTAACGATTTCTGACATGAAATACATGAAAGATTTGTTTTATGTTTGAACGAGGA

Pyrosequencing: T_annealing_ = 51°C

Biotinylated primer: AAATCCCCAAGGGCCTGT

Sequencing primer: ACCGAAGCATTAGGC

***FBA***, Fructose-bisphosphate aldolase (PGSC0003DMG400030565)

Chr05:3708720..3708900

Solcap_c2_11924 c2_11925

ccattgatgaatcgaatgcaactgccggaaagagact[g/a]gc[g/a]tcaattggtctggacaacacagaagcaaacagacaagcttaccgccaactcttgttgaccactcctggtctaggtgattacatctctggatccattctattcgaagagacacttttccagtccactaccgatgggaagaa

Pyrosequencing: T_annealing_ = 57°C

Biotinylated primer: ccattgatgaatcgaatgcaa

Sequencing primer: tgtgttgtccagaccaattga

***CP12-1****,* Chloroplast protein 12, Calvin cycle (PGSC0003DMG400007286)

chr06:195582..195435

ccgattcccctaaaattcaaccgttgaaattcccaaccctaaacagtccatggaagaaatca

solcap_c2_54011

ggtcaattc[a/g]gttacgggaggatgtatgtagtagtacccagagtagttccggataagaagctatctgatttagttgcggaaagtg

Pyrosequencing: T_annealing_ = 56°C

Biotinylated primer: ccgattcccctaaaattcaacc

Sequencing primer: catacatcctcccgtaa

***Mak,*** protein kinase (PGSC0003DMG400020173)

Chr06:58207302..58207970

CCAACTAATGAAAGATCGGCAAAGACCTTTCCTAGAGGAAGAGATTCGAGGATTGATGTCTC

.7407

AGGTGTTGCAAGGACTTGCCCATATGCATAAAAATGGTTACTT(T/C)CATCGGGACTTGAA

.7462 .7475

ACCTGGTATTGAAATGTCATGTGGTCTATTTACTATCTTT(A/T)TTTATGGAAGTG(A/G)

.7485 .7490

GAAACTAAA(G/T)TTAC(A/G)CTTGTTCTGGCTCAGAGAATTTACTGGTGACGAATGACG

.7575.7576

TAATTAAAATTGCTGACTTTGGGTTGGCTCGTGAAGTGTCTTCAT(T/C)(G/A)CCTCCTT

.7588) .7597 .7634

TCAC(T/C)GACTATGT(T/A)TCAACTCGTTGGTGAGTTGTCTTCTCTAGTTTGGTG(T/C

.7637 .7661.7662 .7677

)AT(C/T)CTTTGTTTCCAAGTGCTTGCAAA(A/T)(T/A/G)TTTTGCCTCTGATG(T/C)

.7681 .7687 .7698 .7724

AAG(G/A)ATGTT(C/T)TAATGTCTTT(T/A)AAAGGTACCGAGCACCAGAAGTTTT(G/A

.7736

)TTGCAATCTTC(A/G)TCATACACACCTGCTATCGGTATGCACTGTCAGTACATTCTTGCA

.7813 .7832

ATTTCAGAAGCTTCTTGTGTGGCACACTGGA(T/A)CATTTTTTCATTTGACAC(T/C)AAT

.7839 .7884

AGG(T/C)TTCAGTATGTTTAGAAACTCAAGCTAGCATCCAGAAATTTTGTT(A/C)TTGAA

.7911 .7928

TCTTGACGGCTGTTGCAGCTA(G/A)TTTGTTACTTGTCCTT(A/G)TATACTTGTAAATCT

AAGTACATTGCGCAGCAGTATATTTCG

Amplicon sequencing: T_annealing_ = 59°C

***QUA1****,* Glycosyltransferase QUASIMODO1, PGSC0003DMG400020103

chr06:58259096..58259874

attcctccgtgagcttctccctcctccaagcatccaaatcaaagaagttcattccataagcc

.9166

catgcaca[c/t]gctttggggttgaacttagacttgatcaaaggatgagagaaattcatgtattgtgcataacgatgaaatgaaccaaaacatgtctccacagctccattcaccttacca

.9275(solcap:c2_9204)

[c/t]ccatatctatcttccatagacctgtcaaatccttctgaacaactatatcatcgtccaagaacaatatcctgtgcaacttggggtacaattctggtaaatagaatctaatgtggttcaat

.9409

atggagagatactt[c/t]gggtttctgaatttcatattagttgtatcctttgttgcatttt

.9502

cgagcttattctcgaagtaaaactgctgaagctttgcagattcaagctg[c/t]ttaagtac

.9535(solcap_c2_9203) .9547 .9550

cggaacataagaagaattcaggaa[g/c]ttataatcctc[g/a]ac[c/a]gccttcactt

.9583

cgataaaagccccattatattc[t/c]ttcatcttgaacataacttgcatagccccaagatt

.9652

cattttatcagtcaccacgtgaaacacatgctt[c/t]gatgggtcattcgagtttttaactgctgaattcaccacaactgaagcagcaagcacattgtcagagaagattgcataatgataaaggctaggatcctcaaattctggtggagtaggcttcccatcatctgtatacttatcaggatgagtgattcgctcatccattaacctcattgccaaacaatgcacgctcttcgggatagacttggcagcaatcaagctc

Amplicon sequencing: T_annealing_ = 56°C

***PPR,*** Pentatricopeptide repeat-containing protein (PGSC0003DMG400017649)

Chr09:44067669..44066772

TTGCCCCGAGAAGGCTTAAATTTATTGGCTAAGATGTATTCTGGAGAAGAAAGAGATAGGCT

AGAAGGAAACTGCTTGACCTTTGTGGTAGCTCTTGAAGCATGTTCCCATCTAACAGATTTGG

ACAAAGGAAAGCAAATACATGCAAAAATTATTAGAGAGTTGCCGGATGCGGATGACAATGTA

GCTGTTGGAACAGCTTTAGTTGACATGTATTCAAAATCAGGTCACTTGTGTTACACATTACG

ACTTTTTGATGCGATGGAAGAGAAAAATGTTGTTTCATGGACTTCTGCAATCATGGGATTTG

CTGTTCATGGATTTGCTTTCCAAGCCCTTGAACTTTTTCAGCGTATGGTAAACATGGGAATT

AATCCCAATGAAGTGACATTTACTGCAGTACTAACTGCTTGCCGTCATTGTGGTTTAGTAGA

.7215 .7212 .7207 .7190

TGAGGGAATGCAATATTTTA(C/T)GC(T/A)AATG(T/A)GGAAGCAGTATGGTTT(G/C)

.7189 .7181 .7154 .7145

(A/G)CCCCTGA(T/C)GAAGAACATTATACATGTCTAATTGA(C/T)TTATTGGG(A/G)C

.7083

GTAATGGAAGGCTTGAGGAAGCATGGCATTTGGTGGAGGGAATGGAAGAGAACCATCTAA(A

.7077 .7067 .7064 .7060 .7053

/G)TGATG(G/A)GTGCTCTAC(A/T)GG(T/C)ACC(A/G)TTTGGG(C/G)TGCACTTCT

.7037 .7031 .6998

AGGGGC(C/T)TGTCA(G/A)TTGTATGAAAACGTTGAAATTGGAAAGAAGGT(A/G)GCTG

AAAAGCTGTCGGAAAAGGAGATACTGATATCTACTGCTTCTATTGCGCTATCTAATGTTTAT

GCAGCAGCTGGAATGTGGAATGAAGTGTACAGAGTGAGAGAGAGTTGGAGAAAAAAAGGTCA

TGCTGATGGAGAGCCTGGTCTTAGCCGCATATGTACACAACCCTATTAATACGAAATTCTTA

GACACAGTGTTGTTGCCCTTTTATGTAAATGGTGAG

Amplicon sequencing: T_annealing_ = 55°C

***RP60S***, 60S acidic ribosomal protein PO, PGSC0003DMG400029622

chr09:58375196..58375302

solcap_c2_3063

gcaagggattcgtaagggtct[c/t]cgaggtgactctgtcgtacttatggggaaaaatactatgatgaaaaggtccgttaggatccatgcgaaaagtaccggaaacgatg

Pyrosequencing: T_annealing_ = 56°C

Biotinylated primer: catcgtttccggtacttttcg

Sequencing primer: gcaagggattcgtaagggtct

***CIS***, Citrate synthase, PGSC0003DMG400007797

Chr12:1324412..1324922

.4470

Ggatgcgtcactatatgcctctcaaagaacgatcaccacattccgaagcagacaagct[c/t

.4476(c2_25372).4479 .4485 .4495

]ggtca[c/t]gt[c/g]tccgt[c/t]tccaatgcc[a/t]ccaaacgacgattggc

.4512 .4545

[t/c]ggttcaggggcctaaggcttccttcaaggttc[a/t]cttatatttaatataagaaaaataaatgatatttttttcttccccatgagaagtaaacttgtcttgaacatggttagattctcacacacacaaagcagatgtattcggatgaatttttgtgtctccgtttcccaaaataataagcttttagtcttcacaagtaacaaatttgcatcctccccatatttcctagtcctacaatgatatggagtgtacctaattatagtaacttttaacttgaacctaatccaactcaaaaaagcctttatacgatcgtttatatttcattctttaggtcctcacttttcacttactatcatcaatcacaatcaaatattcatggaagattacaactatattgtagcatgcatcccagg

Amplicon sequencing: T_annealing_ = 56°C
